# A rechargeable aqueous manganese-ion battery based on intercalation chemistry

**DOI:** 10.1038/s41467-021-27313-5

**Published:** 2021-11-30

**Authors:** Songshan Bi, Shuai Wang, Fang Yue, Zhiwei Tie, Zhiqiang Niu

**Affiliations:** Key Laboratory of Advanced Energy Materials Chemistry (Ministry of Education), College of Chemistry, Nankai University, Tianjin, 300071 P. R. China

**Keywords:** Materials for energy and catalysis, Structural materials, Batteries, Batteries

## Abstract

Aqueous rechargeable metal batteries are intrinsically safe due to the utilization of low-cost and non-flammable water-based electrolyte solutions. However, the discharge voltages of these electrochemical energy storage systems are often limited, thus, resulting in unsatisfactory energy density. Therefore, it is of paramount importance to investigate alternative aqueous metal battery systems to improve the discharge voltage. Herein, we report reversible manganese-ion intercalation chemistry in an aqueous electrolyte solution, where inorganic and organic compounds act as positive electrode active materials for Mn^2+^ storage when coupled with a Mn/carbon composite negative electrode. In one case, the layered Mn_0.18_V_2_O_5_·nH_2_O inorganic cathode demonstrates fast and reversible Mn^2+^ insertion/extraction due to the large lattice spacing, thus, enabling adequate power performances and stable cycling behavior. In the other case, the tetrachloro-1,4-benzoquinone organic cathode molecules undergo enolization during charge/discharge processes, thus, contributing to achieving a stable cell discharge plateau at about 1.37 V. Interestingly, the low redox potential of the Mn/Mn^2+^ redox couple vs. standard hydrogen electrode (i.e., −1.19 V) enables the production of aqueous manganese metal cells with operational voltages higher than their zinc metal counterparts.

## Abstract (web summary)

Multivalent metal batteries are considered a viable alternative to Li-ion batteries. Here, the authors report a novel aqueous battery system when manganese ions are shuttled between an Mn metal/carbon composite anode and inorganic or organic cathodes.

## Introduction

**Fig. 1:**
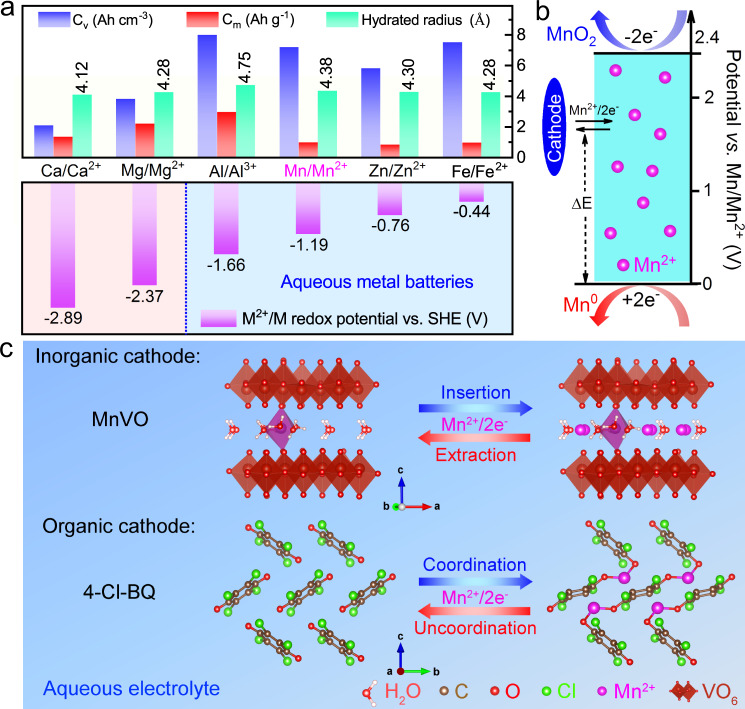
Properties of Mn^2+^ ions in aqueous electrolytes. **a** Comparison of the theoretical capacity, hydrated ionic radius, and M/M^2+^ redox potential vs. SHE of various multivalent metals, including Ca, Mg, Al, Mn, Zn, and Fe. **b** The stable voltage window of Mn^2+^ ions. **c** Schematic diagram of different Mn^2+^ ion reaction processes in inorganic (MnVO) and organic (4-Cl-BQ) cathodes.

Aqueous rechargeable batteries are intrinsically safe due to the utilization of low-cost and nonflammable water-based electrolytes, thereby displaying robustness and cost advantages over competing for commercial lithium-ion batteries with volatile organic electrolytes^1–5^. Among various aqueous rechargeable batteries, those that pair metal anodes with various cathode materials are usually competitive in energy density because metal anodes provide much higher capacity and lower reduction potential than intercalation anodes. Up to now, Zn, Fe, and Al have been directly utilized as the anodes of aqueous rechargeable batteries since they are stable in aqueous electrolytes and possess high theoretical specific capacity^6–10^. Whereas the reduction potentials vs. standard hydrogen electrode (SHE) of Zn and Fe (Zn: −0.76 V, and Fe: −0.44 V) are high (Fig. 1a), resulting in low operating voltages of their corresponding aqueous batteries^11,12^. Compared with Zn and Fe, Al possesses lower theoretical reduction potential (−1.66 V vs. SHE). However, in fact, the practical reduction potential at Al anode is only around −0.7 V vs. SHE in aqueous Al-ion batteries (AIBs), which would be ascribed to the large polarization of Al anodes^13–16^. As a result, the discharge voltages of aqueous AIBs are also limited^17,18^. In addition, the high charge density of Al^3+^ and the oxide layer on Al anode often result in unsatisfactory rate capability and poor cycling stability of aqueous AIBs^19^. Therefore, it is of great significance to explore other aqueous metal battery systems with both excellent electrochemical performance and promising futures in practical applications.

Manganese is the second most abundant transition metal near the Earth’s surface. Owing to its high electronegativity, Mn metal also exhibits less reactivity and therefore reliable safety to be handled in water^20,21^. Furthermore, in theory, Mn possesses a low reduction potential (−1.19 V vs. SHE) and high capacity (7250 mAh cm^−3^ and 976 mAh g^−1^, based on two-electron transfer reactions)^22^. These properties of Mn make it a natural choice to act as the anode material in high-voltage aqueous batteries, where Mn ions will be employed as charge carriers. According to the standard electrode potentials, Mn ions could remain +2 valence in a wide voltage window (~2.4 V) (Fig. 1b), which will ensure the stable Mn^2+^ ion transportation in electrolytes with a wide working voltage range. Therefore, rechargeable aqueous Mn-ion batteries (MIBs) could be feasible in principle and provide cost-effectiveness and considerably high energy density. However, to our best knowledge, there is hardly any report about Mn^2+^ ion charge carrier in battery research. Divalent Mn^2+^ ion holds a larger solvated ionic radius in aqueous electrolytes than the cases of most divalent ions, such as Ca^2+^, Mg^2+^, Zn^2+^, and Fe^2+^, indicating more difficulty of intercalating Mn^2+^ ions in common host materials. As a result, the implementation of aqueous MIBs hinges on the design of Mn^2+^ ion host materials with large interspacing channels and weak bond strengths between intercalated Mn^2+^ ions and host frameworks.

In this work, we reported the reversible Mn^2+^ intercalation chemistry in inorganic and organic compounds in aqueous battery systems (Fig. 1c). The Mn_0.18_V_2_O_5_·*n*H_2_O (MnVO) cathode is first selected as the host material of Mn^2+^ ions. Its large lattice spacing enables a fast and reversible Mn^2+^ ion insertion/extraction process, leading to high power density and stable cycling performance. In addition to MnVO, the quinone molecule, tetrachloro-1,4-benzoquinone (4-Cl-BQ), undergoes the enolization redox chemistry in the charge/discharge process with a stable discharge plateau. More impressively, the low redox potential vs. SHE of Mn/Mn^2+^ endows the resultant aqueous Mn metal batteries with high operating voltages. These results indicate the feasibility of Mn^2+^ ion intercalation in different cathodes as well as the promising future of aqueous MIBs.

## Results

### Design toward aqueous MIBs

Towards rechargeable aqueous MIBs, Mn^2+^ ion dissociation from aqueous electrolytes and solid-state ion diffusion in cathodes are two essential steps during the charge/discharge process^23^. Therefore, it is of great importance to explore suitable electrolytes and cathode materials of MIBs. In common aqueous metal-ion battery systems, electrolytes are usually obtained via dissolving metal salts into water, where metal cations act as charge carriers. In addition to metal cations, anions in electrolytes also play an important role in the electrochemical performance of aqueous metal-ion batteries since their redox activity would affect the electrochemical window of electrolytes and the compatibility with electrodes^24^. Moreover, the coordination capability of anions with metal cations also determines the metal ion intercalation process. MnCl_2_, Mn(NO_3_)_2_, MnSO_4_, and Mn(CF_3_SO_3_)_2_ are the common manganese salts, which could be employed as the aqueous electrolytes via dissolving them into water. To understand the electrochemical window of MIBs with these aqueous electrolytes, the cells were assembled, where Mn/C composite and stainless steel served as anode and cathode, respectively. It is noted that the electrolyte decomposition will occur after 1.7 and 1.5 V in 1 M MnCl_2_ and Mn(NO_3_)_2_ aqueous electrolytes, respectively (Supplementary Fig. 1), which was due to that Cl^−^ and NO_3_^−^ possess high redox activity^24,25^. Therefore, MnCl_2_ and Mn(NO_3_)_2_ aqueous electrolytes are not feasible for aqueous MIBs. In contrast, SO_4_^2−^ and CF_3_SO_3_^−^ possess stable structures^24^, indicating the high electrochemical stability and compatibility with electrodes. The electrochemical window could reach ~2.0 V within MnO_2_ deposition and H_2_ evolution potentials in these two electrolytes (Supplementary Fig. 2). Moreover, Mn metal could be electrodeposited on stainless steel at a low potential (Supplementary Fig. 3), indicating the feasibility of MnSO_4_ and Mn(CF_3_SO_3_)_2_ solutions to act as the electrolytes of aqueous MIBs. The contact angles of MnSO_4_ and Mn(CF_3_SO_3_)_2_ electrolytes were measured. Electrolyte droplets (~5 μL) were dropped on the surface of the MnVO cathode and then let it stand for 5 s. Compared with MnSO_4_ electrolyte, Mn(CF_3_SO_3_)_2_ electrolyte exhibits greater hydrophilicity on cathode due to the existence of CF_3_ groups (Supplementary Figs. 4 and 5), which will be beneficial to the infiltration of electrolyte into active materials. Besides, the bulky CF_3_SO_3_^−^ anions could also reduce the solvation effect of metal ions, facilitating metal ion transportation and charge transfer^25,26^. As a result, Mn(CF_3_SO_3_)_2_ electrolytes would be the better choice to act as the electrolyte of aqueous MIBs.

Apart from electrolytes, cathode materials also determine the ion insertion thermodynamics and kinetics in aqueous batteries. Different from other divalent metal ions, Mn^2+^ ions possess a large solvated ionic radius and enhanced interaction with frameworks of host materials, which will limit the insertion/extraction of Mn^2+^ ions in common host materials. Among cathode candidates, metal oxides are perspective due to their low cost and high valence of metal centers^27^. In consideration of the multivalence of vanadium and large lattice spacing, layered vanadium-based compounds exhibit promising to function as Mn^2+^ ion host materials. Furthermore, the interlayer spacing of layered vanadium-based compounds can be adjusted by introducing metal ions and structural water molecules^28–30^. The introduced metal ions in the interlayers can act as pillars to enhance their structural stability during the charge/discharge process. The structural water molecules between V_*x*_O_*y*_ layers could reduce the effective charge of metal ions by solvation, accelerating metal ion diffusion^31^. Therefore, in our case, we designed an Mn^2+^ ion pre-intercalated hydrated vanadium oxide (MnVO) as the cathode material of aqueous MIBs to accommodate the Mn^2+^ ion charge carriers (Supplementary Figs. 6–9 and Supplementary Note 1).

In addition to layered inorganic materials, organic compounds can also serve as the cathode materials of aqueous metal-ion batteries by the coordination/uncoordination chemistry, which is different from the insertion/extraction mechanism in inorganic compounds and usually not governed by the radius and charge of charge carriers^32^. Furthermore, organic materials are sustainable and their structures can be designed flexibly, which offers the potential for the concomitant binding with Mn^2+^ ions^33^. Therefore, aqueous Mn||organic cells could be realized by designing appropriate organic compounds, which will broaden the horizons of MIBs. Quinones exhibit a highly reversible transition process between C = O and C–O^−^ groups during electrochemical redox reactions^34,35^. Furthermore, the introduction of electron-withdrawing groups (such as -CN, -F, -Cl, and -Br) into quinones could improve their operating voltages^33^. Thus, in our case, 4-Cl-BQ was selected as the cathode material to assemble an aqueous Mn||organic cell.

### Mn^2+^ insertion in MnVO cathodes

**Fig. 2:**
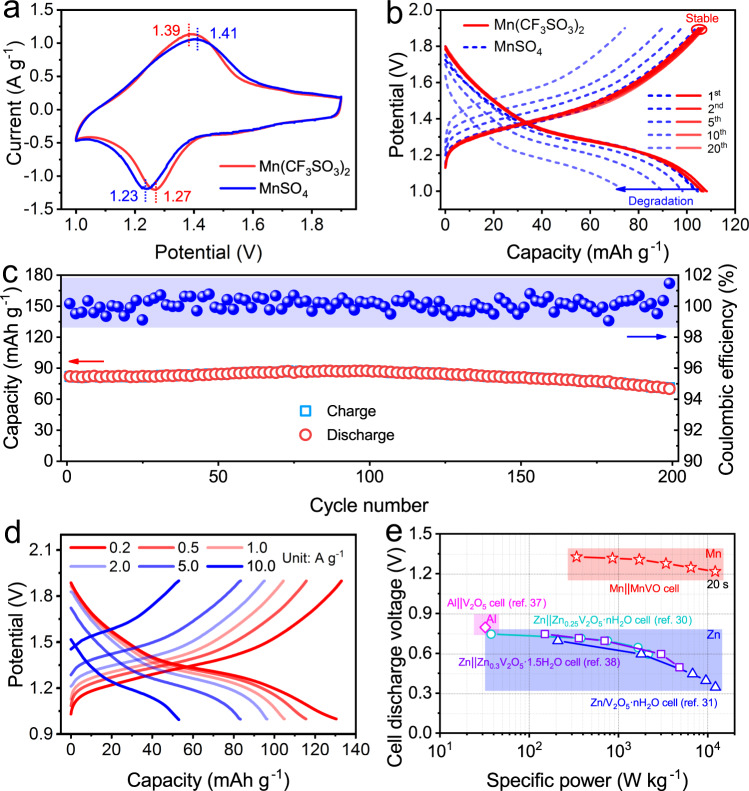
Electrochemical performance of the Mn||MnVO cells. **a** CV curves at 1.0 mV s^−1^ and **b** GCD curves at 1.0 A g^−1^ in 1 M MnSO_4_ and Mn(CF_3_SO_3_)_2_ electrolytes. **c** Cycling performance at 5.0 A g^−1^ in 1 M Mn(CF_3_SO_3_)_2_ electrolyte. **d** GCD curves at different specific currents. **e** Comparison of the cell discharge voltage and specific power between various vanadium-based aqueous metal cells.

To understand the Mn^2+^ ion insertion in MnVO, Mn||MnVO coin cells were assembled with Mn/C anodes, MnVO cathodes, and aqueous Mn(CF_3_SO_3_)_2_ electrolytes (Supplementary Fig. 10). The Mn||MnVO cell displays a pair of reversible reduction/oxidation peaks located at 1.27/1.39 V in their cyclic voltammetry (CV) curves (Fig. 2a), which could be ascribed to a one-step Mn^2+^ ion insertion/extraction process. Moreover, their CV curves exhibit similar shapes at the initial three cycles (Supplementary Fig. 11a), revealing the highly reversible redox behaviors. Their galvanostatic charge/discharge (GCD) curves are consistent well with the CV results. The Mn||MnVO cell delivers an average discharge voltage of around 1.33 V and a capacity of 133.7 mAh g^−1^ at 0.2 A g^−1^ (Supplementary Fig. 11b), corresponding to a ~1.1 electron redox process. In addition, there is nearly no deviation in the GCD curves at the initial 20 cycles (Fig. 2b), indicating that the Mn||MnVO cell shows stable Mn^2+^ ion insertion/extraction performance. Moreover, capacity retention of 86.7% was achieved at a specific current of 5.0 A g^−1^ after 200 cycles (Fig. 2c). A similar reaction process was also observed in Mn||MnVO cells with aqueous MnSO_4_ electrolyte. However, the Mn||MnVO cell exhibits a larger polarization voltage in MnSO_4_ electrolyte than that in Mn(CF_3_SO_3_)_2_ electrolyte. Besides, in the case of MnSO_4_ electrolyte, the capacity undergoes a rapid decay at cycling process (see Fig. 2b). The performance of the Mn||MnVO cells with Mn(CF_3_SO_3_)_2_ electrolyte is ascribed to that the CF_3_SO_3_^−^ anions could decrease the solvation effect of Mn^2+^ ions in aqueous solution, facilitating Mn^2+^ ions insertion/extraction process (Supplementary Fig. 12 and Supplementary Table 1). Therefore, Mn(CF_3_SO_3_)_2_ electrolyte was further selected for the Mn^2+^ ion reaction kinetics and mechanism analysis in MnVO electrodes.

The insertion/extraction kinetics of Mn^2+^ ions in the MnVO electrode could be understood by their CV curves at different scan rates. The reduction/oxidation peaks in CV curves are still detectable at high scan rates (Supplementary Fig. 13a). The relationship between peak currents (*i*) and scan rates (*v*) could be written as follows^36^:

1 $$i=a{v}^{b}$$

which could be rearranged to

2 $${{{{{\rm{log }}}}}}\left(i\right)=b\,{{{{{\rm{log }}}}}}\,\left(v\right)+{{{{{\rm{log }}}}}}\,\left(a\right)$$

where *b* is the slope of log(*i*) vs. log(*v*) curve. The *b* value approaching to 0.5 indicates an ionic diffusion-controlled electrochemical process. When *b* value reaches 1, the charge/discharge process is determined by pseudocapacitance. By fitting the plots of log(*i*) vs. log(*v*), the *b* values for oxidation and reduction peaks are fitted as 0.68 and 0.71 (Supplementary Fig. 13b), suggesting that the charge/discharge process in Mn||MnVO cells is controlled by ionic diffusion and pseudocapacitance synergistically. This characteristic contributes to the rate capability performance of the Mn||MnVO cells, where a considerable capacity of 83.3 mAh g^−1^ could still be obtained even at a specific current of 5.0 A g^−1^ (Fig. 2d and Supplementary Fig. 14). The typical discharge plateau is maintained at 1.25 V, which is higher than the cases of the state-of-the-art vanadium-based aqueous metal batteries, including Al||V_2_O_5_ cells (0.8 V) and Zn||Zn_*x*_V_2_O_5_·*n*H_2_O cells (<0.75 V)^30,31,37,38^. Furthermore, the Mn||MnVO cells could also deliver a specific power of 12.1 kW kg^−1^ at 10.0 A g^−1^ (Fig. 2e), which is comparable with the high-rate aqueous Zn||V_2_O_5_·*n*H_2_O cells (12.2 kW kg^−1^) and much higher than that of the aqueous Al||V_2_O_5_ cells (<50 W kg^−1^)^31,37^. At such specific power, the charging process was fully completed in 20 s with a specific energy of 64.8 Wh kg^−1^.

### Ex situ measurements and analyses of the MnVO-based inorganic electrode

**Fig. 3:**
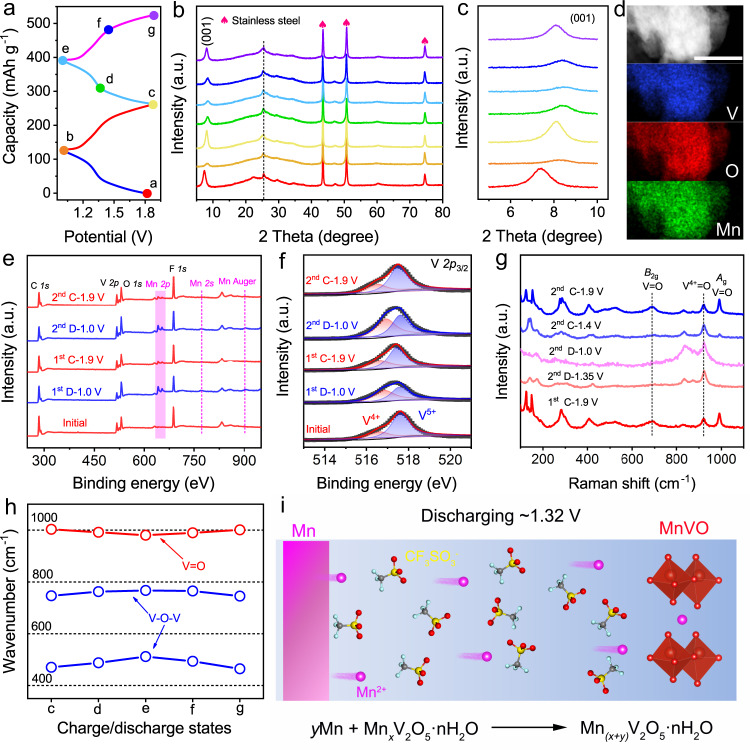
Mn^2+^ insertion mechanism in MnVO electrodes. **a** The initial two GCD curves at 0.2 A g^−1^. **b** XRD patterns at different charge/discharge states. **c** An enlarged view of the (001) reflection from the XRD patterns in (**b**). **d** TEM element mapping in fully discharged electrodes. Scale bar: 200 nm. XPS spectra of **e** full-spectrum and **f** V 2*p*_3/2_ at fully discharged/charged states. **g** Raman and **h** FTIR spectra at the selected charge/discharge states. **i** Schematic diagram of the discharging mechanism of Mn||MnVO cells.

The energy storage process plays an important role in the electrochemical performance of Mn||MnVO cells. Thus, various measurements, including X-ray diffraction (XRD), transmission electron microscopy (TEM), X-ray photoelectron spectroscopy (XPS), Raman and Fourier transform infrared (FTIR), were conducted on MnVO electrodes at selected states in the charge/discharge process to understand the energy storage mechanism (Fig. 3a). In XRD spectra, compared to a pristine state, no new reflection is observed and only the reflection of the (001) plane shifts slightly toward a high degree during the discharge process (Fig. 3b, c), suggesting the decrease of the corresponding interlayer spacing due to the Mn^2+^ ion insertion in the interlayer of MnVO (Supplementary Fig. 15). The Mn^2+^ ion insertion/extraction mechanism in MnVO cathodes is further demonstrated by energy-dispersive X-ray (EDX) spectrum results of TEM, in which the molar ratio of Mn/V elements increases in fully discharged electrodes and then decreases in recharged case (Fig. 3d and Supplementary Figs. 16 and 17). Moreover, in XPS spectra, the peaks of Mn 2*p*, Mn 2*s*, and Mn Auger are significantly strengthened in MnVO electrodes at a fully discharged state (Fig. 3e). Reversibly, during the subsequent charging process, the intensities of these peaks are weakened. Therefore, XPS results also suggest the reversible Mn^2+^ insertion/extraction in MnVO cathodes. Inductively coupled plasma atomic emission spectroscopy (ICP-AES) shows that the molar ratios of Mn and V in the discharged and charged products are 0.385:1 (first discharge at 1.0 V) and 0.097:1 (first charge at 1.9 V), respectively (Supplementary Fig. 18 and Supplementary Table 2). Therefore, in the charging process, the amount of the extracted Mn^2+^ is 0.576 in per formula unit of MnVO, corresponding to a ~1.15 electron redox process (at 0.2 A g^−1^). It agrees well with the charge/discharge capacities.

The insertion/extraction of Mn^2+^ ion will lead to the valence change of vanadium in MnVO cathodes, as illustrated in V 2*p* XPS spectra at different charge/discharge states (Fig. 3f). At a fully discharged state, the peak of V 2*p*_3/2_ shifts to lower bonding energy, corresponding to the reduction of vanadium. After recharging, it nearly recovers to the initial state since vanadium is oxidized along with the Mn^2+^ extraction. The vanadium redox reaction can also be investigated via Raman and FTIR measurements and analyses. During Mn^2+^ insertion, the Raman peaks between 850 and 950 cm^−1^ appear and enhance gradually (Fig. 3g), which is attributed to the V^5+^/V^4+^ transition^39^. In FTIR spectra, the peaks of V=O and V–O–V bonds shift to lower and higher wavenumber, respectively (Fig. 3h), suggesting the weakening of V=O bonds and strengthening of V–O–V bonds^40,41^. In the subsequent recharging process, these peaks almost return to the initial state, revealing the high reversibility of the V^5+^/V^4+^ transition. In addition, there is no peak emerging at around 3500 cm^−1^ (Supplementary Fig. 19), which is assigned to –OH bend^37^, indicating that the protons in the electrolyte would not insert into the MnVO cathode in our case. Thus, the insertion/extraction of Mn^2+^ ions is realized in Mn||organic cell (Fig. 3i).

### Mn^2+^ coordination in organic cathodes

**Fig. 4:**
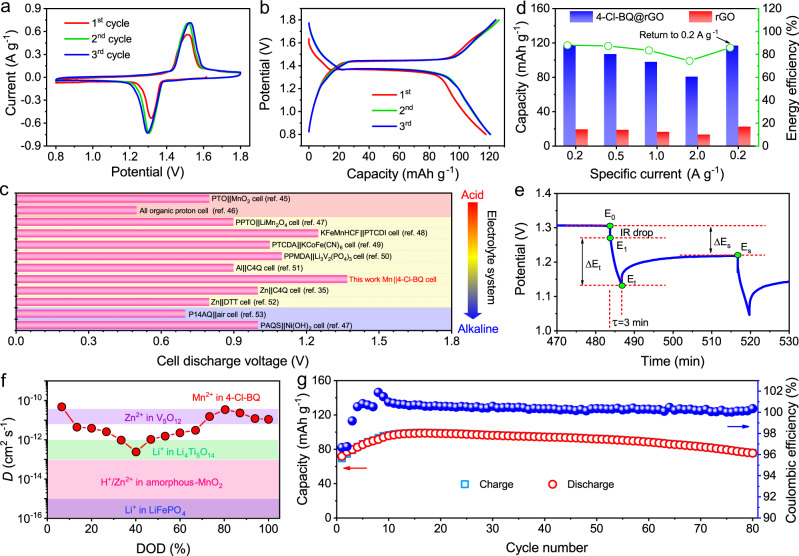
Electrochemical performance of the Mn||4-Cl-BQ cells. **a** CV curves at 0.2 mV s^−1^. **b** GCD curves at 0.2 A g^−1^. **c** Comparison of the cell discharge voltages between different aqueous quinone-based cells in acid, mild and alkaline electrolytes. **d** Capacities of rGO and 4-Cl-BQ@rGO composites at different specific currents. The right *y*-axis is the energy efficiency of Mn||4-Cl-BQ cells. **e** Selected steps of the GITT curve at 0.2 A g^−1^ during discharging, in which the key parameters for diffusion coefficient calculation are marked. **f** The calculated Mn^2+^ ion diffusion coefficient during discharging and the comparison with other battery systems. **g** Cycling performance at 1.0 A g^−1^.

Organic compounds are also promising Mn^2+^ ion host materials in consideration of their potentially sustainable production and structural variability^32^. To illustrate the feasibility of Mn^2+^ ion coordination in organic materials, 4-Cl-BQ was employed as the cathode material of aqueous Mn||organic cells due to its high capacity and flat cell discharge voltage in aqueous Zn-ion cells^42^. In general, organic materials suffer from poor electronic conductivity, which would limit their rate capability. Furthermore, the discharged products of quinones always face serious dissolution in aqueous electrolytes, degrading cycling stability^43,44^. Therefore, 4-Cl-BQ molecules were introduced into reduced graphene oxide (rGO) foams, which possess conductive and continuous networks (Supplementary Figs. 20–23 and Supplementary Note 2). Since the resultant 4-Cl-BQ@rGO composite foams are conductive and freestanding, they can serve as the cathodes directly, where neither polymer binders nor conductive additives are required. The CV curves of Mn||4-Cl-BQ cell show one pair of redox peaks located at 1.29/1.53 V, indicating one-step coordination/uncoordination reaction during the discharge/charge process (Fig. 4a). Correspondingly, in GCD curves, the Mn||4-Cl-BQ cell delivers a stable discharge plateau at around 1.37 V (0.2 A g^−1^) (Fig. 4b). The discharge voltage is higher than the cases of those reported aqueous batteries with quinone electrodes in various electrolytes (Fig. 4c)^35,45–53^. The higher discharge voltage is attributed to the redox potential of Mn/Mn^2+^ being lower than the cases of intercalation anodes and other metal anodes (Al and Zn) in aqueous batteries.

Apart from high and stable discharge voltage, the aqueous Mn||4-Cl-BQ cells also exhibit good cycling rate performances and energy efficiency (Fig. 4d). The cell could deliver a capacity of around 80.6 mAh g^−1^ at 2.0 A g^−1^, maintaining 68.5% of that at 0.2 A g^−1^ (Supplementary Fig. 24), which suggests the fast kinetics of Mn^2+^ ion coordination/uncoordination in 4-Cl-BQ molecules. The galvanostatic intermittent titration technique (GITT) was further employed to understand the diffusion coefficient of Mn^2+^ ions in 4-Cl-BQ@rGO composite cathodes. The Mn||4-Cl-BQ cell was discharged at 0.2 A g^−1^ with an interval of 3 min and then relaxed for 30 min to allow the voltage to reach equilibrium (Supplementary Fig. 25). The *D*_ion_ was calculated according to the equation^54^:

3 $${D}_{{{{{{\rm{ion}}}}}}}=\frac{4}{{{{{{\rm{\pi }}}}}}t}{\left(\frac{m{V}_{m}}{{MS}}\right)}^{2}{\left(\frac{{\triangle E}_{S}}{{\triangle E}_{\tau }}\right)}^{2}\,$$

in which *τ* is the duration time (s) of the current pulse and *m* is the weight of 4-Cl-BQ (g). *M* and *V*_*m*_ are the corresponding molar mass (g mol^−1^) and volume (cm^3^ mol^−1^) of 4-Cl-BQ, respectively. *S* is the contact area (cm^2^) between electrode and electrolyte. Δ*E*_*s*_ and Δ*E*_*τ*_ are related to the change of discharge voltage and steady-state voltage for the corresponding steps (Fig. 4e). GITT result shows that the diffusion coefficient of Mn^2+^ ions in 4-Cl-BQ@rGO composite cathode is ~10^−12^–10^−10^ cm^2^ s^−1^, which is higher than the Li^+^ ion diffusion coefficients in LiFePO_4_ and Li_4_Ti_5_O_12_ (<10^−12^ cm^2^ s^−1^) (Fig. 4f)^49^. Furthermore, it is comparable with the Zn^2+^ ion diffusion coefficient in V_5_O_12_ and higher than the H^+^/Zn^2+^ ion diffusion coefficient in amorphous MnO_2_^54,55^. In addition, the introduction of rGO foams in cathodes endows the Mn||4-Cl-BQ cells with relatively stable cycling performance (Fig. 4g). Initially, the 4-Cl-BQ@rGO composite cathodes deliver a capacity of 71.9 mAh g^−1^ at 1.0 A g^−1^. After 80 cycles, the capacity still remains at 75.6 mAh g^−1^.

### Ex situ measurements and analyses of the 4-Cl-BQ-based organic electrode

**Fig. 5:**
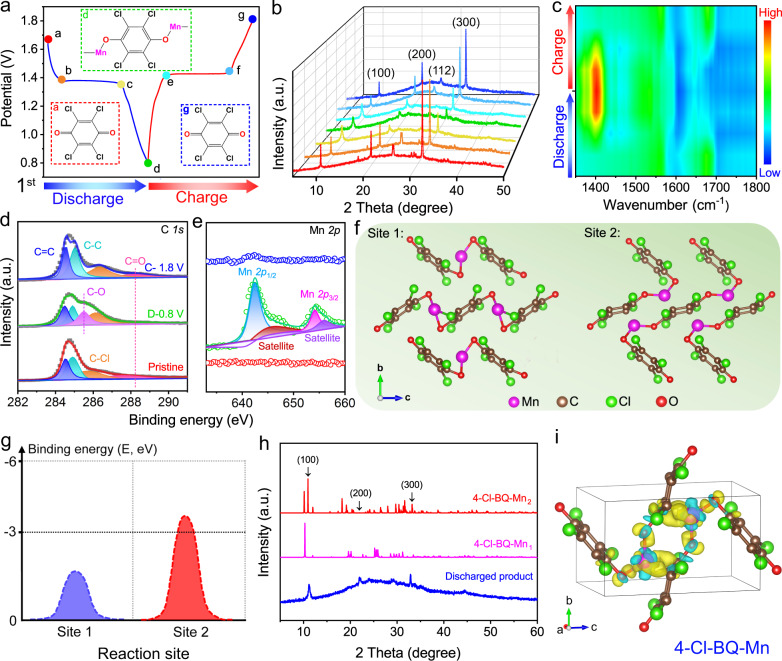
Mn^2+^ ion coordination mechanism in 4-Cl-BQ electrodes. **a** The first GCD curve at 50 mA g^−1^. Inserts are the schematic diagrams of 4-Cl-BQ molecules at different discharge/charge states. **b** XRD patterns, **c** FTIR, XPS spectra of **d** C 1*s* and **e** Mn 2*p* at different discharge/charge states. **f** Schematic diagram and **g** calculated binding energy of two possible coordination sites of Mn^2+^ ions in 4-Cl-BQ crystals. **h** The predicted XRD patterns of two possible coordination sites and the XRD pattern of the discharged product. **i** Differential charge densities of Mn^2+^ ion coordinated 4-Cl-BQ crystals. Blue and yellow indicate electron accumulation and depletion, respectively.

Different from the insertion/extraction energy storage mechanism in inorganic materials, 4-Cl-BQ molecules often exhibit the coordination/uncoordination chemistry in the discharge/charge process. In our case, the energy storage mechanism of the 4-Cl-BQ cathode was investigated via a series of ex situ tests, including XRD, XPS, and FTIR at the selected states in the first cycle (Fig. 5a). As shown in XRD patterns, the sharp reflections at 21.7°, 26.6°, and 32.7° belonging to (200), (112) and (300) planes, get weakened during discharging (Fig. 5b), which is due to that the ion coordination process degrades the crystallinity of 4-Cl-BQ. The 4-Cl-BQ undergoes the enolization redox chemistry in the charge/discharge process, as suggested by FTIR spectra. During the discharge process, the peak intensity of the C=O group vibration (stretching vibration: 1655 cm^−1^) gradually declines, indicating the reaction between Mn^2+^ ions and C=O groups (Fig. 5c). Furthermore, XPS on C 1*s* of 4-Cl-BQ@rGO composite electrode was further performed to understand the transformation between C=O and C–O^−^ groups (Fig. 5d). Compared with the C 1*s* spectra of the pristine 4-Cl-BQ@rGO composite electrode, the characteristic peak of C=O bonds at 288.1 eV becomes invisible at a fully discharged state. Besides, a new peak at 285.5 eV assigned to C–O bonds emerges. Reversibly, these peaks corresponding to C=O and C–O bonds return to the initial state in recharged electrodes. There is a possible competition between protons and Mn^2+^ ions to react with 4-Cl-BQ molecules during the discharge process due to the coexistence of these two cations in electrolytes. Protons could coordinate with 4-Cl-BQ molecules to form dihydro-p-chloranil (4-Cl-HQ) in the acid electrolyte at discharged state^56^, whereas no characteristic peak of 4-Cl-HQ is observed in the XRD pattern of discharged products (Supplementary Fig. 26), suggesting that protons would not coordinate with 4-Cl-BQ molecules in the electrolyte solution we used. XPS spectra on Mn 2*p* were further conducted to demonstrate whether Mn^2+^ ions in electrolyte coordinate with 4-Cl-BQ molecules. Two peaks belonging to Mn *2p*_1/2_ and Mn *2p*_3/2_ are observed at a fully discharged state (Fig. 5e), which indicates the Mn^2+^ ion coordination. Furthermore, the elemental mapping also demonstrates the existence of Mn in discharged products (Supplementary Figs. 27 and 28). It is consistent with the XPS results. These results imply the Mn^2+^ coordination/uncoordination in 4-Cl-BQ during the discharge/charge process.

At a fully discharged state, one Mn^2+^ ion needs to coordinate with two C–O^−^ groups. However, owing to the long distance between two C–O^−^ groups in one 4-Cl-BQ molecule, Mn^2+^ ion would coordinate with adjacent two 4-Cl-BQ molecules during the discharge process. Thus, the nonsymmetrical crystal structure of 4-Cl-BQ molecules results in two possible coordination sites of Mn^2+^ ions in 4-Cl-BQ crystals: (1) Mn^2+^ ion uptake occurs along the *c-*axis between adjacent 4-Cl-BQ molecules (4-Cl-BQ-Mn_1_); (2) Mn^2+^ ion uptake takes place along the *b* axis between adjacent 4-Cl-BQ molecules (4-Cl-BQ-Mn_2_) (Fig. 5f). To understand where the Mn^2+^ ion coordinates with 4-Cl-BQ molecules at a fully discharged state, theoretical calculations with density functional theory (DFT) were conducted. The XRD pattern of optimized 4-Cl-BQ is consistent with 4-Cl-BQ@rGO electrodes (Supplementary Fig. 29). The calculated results show that the binding energy of 4-Cl-BQ-Mn_2_ is lower than the case of 4-Cl-BQ-Mn_1_ (Fig. 5g). Furthermore, in the simulated XRD pattern of 4-Cl-BQ-Mn_2_, the peaks corresponding to (100), (200), and (300) planes are observed (Fig. 5h), which is consistent with the experimental XRD results. Therefore, 4-Cl-BQ-Mn_2_ is the most likely structure of the discharged product. Owing to the strong electrostatic interactions between Mn^2+^ ions and C–O^−^ groups, the Mn^2+^ ion coordination results in the decrease of the O–O distance between C–O^−^ groups in adjacent 4-Cl-BQ molecules (Supplementary Fig. 30). Furthermore, we calculated the charge density difference of the discharge product (Fig. 5i). Electron deficient region is found around the coordinated Mn atoms, suggesting a significant electron transfer from Mn to 4-Cl-BQ^57^, which contributes to a more stable crystal structure of 4-Cl-BQ after Mn^2+^ coordination.

### Opportunities and challenges of aqueous MIBs

**Fig. 6:**
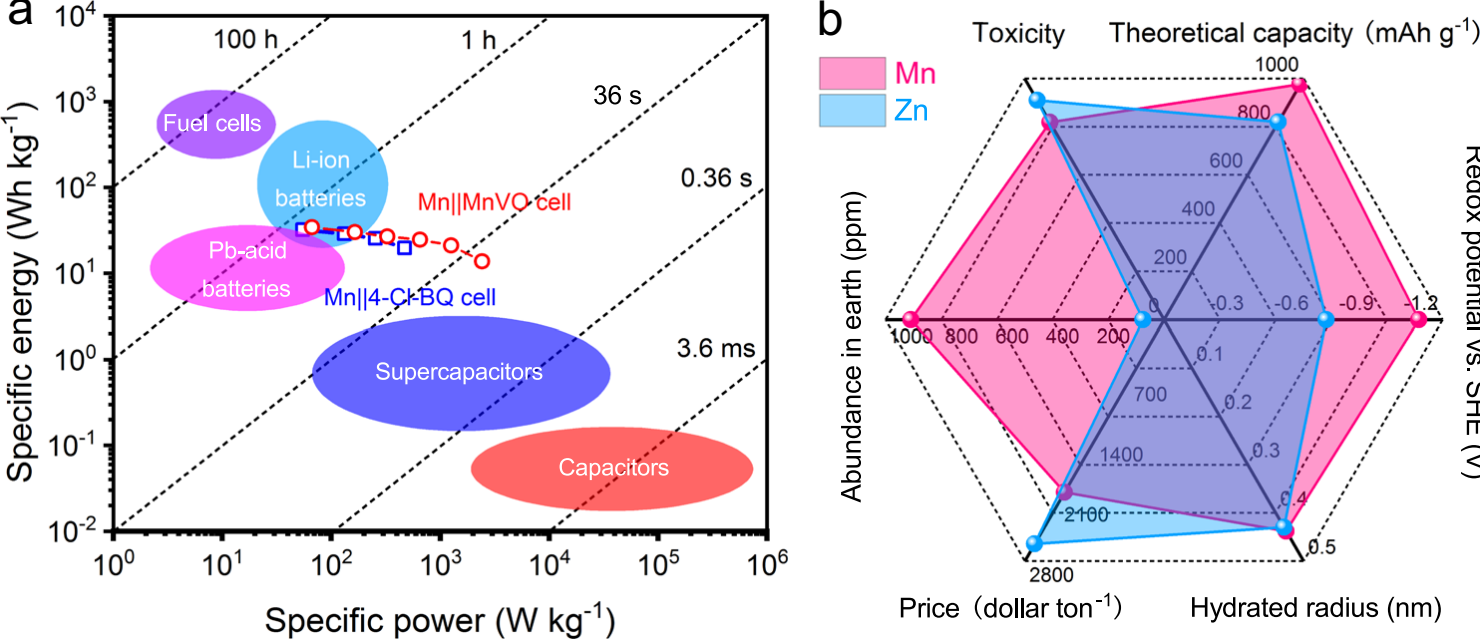
Comparison of aqueous MIBs with other energy storage systems. **a** Ragone plot showing the specific energy and power of the aqueous Mn cells with various commercial energy storage devices^60^. **b** Comparison of the general features between Mn and Zn^6^.

The aqueous MIBs have some advantages over conventional rechargeable energy storage systems. Compared with Pb-acid batteries, the aqueous MIBs show higher specific power (Fig. 6a). Moreover, at the high specific power, they can also deliver higher specific energy than aqueous and non-aqueous supercapacitors. Therefore, aqueous MIBs could be promising for large-scale electrochemical energy storage applications. In contrast with aqueous zinc-ion batteries, the motivations triggering the development of aqueous MIBs are related to the general features of Mn metal. Firstly, Mn metal is more abundant, more inexpensive, and with lower toxicity than Zn metal (Fig. 6b), which is important for evaluating sustainability and practical applications. Besides, aqueous MIBs could deliver higher specific energy when paired with the same redox couples, resulting from the lower standard reduction potential and higher capacity (−1.19 V vs. SHE; 976 mAh g^−1^) of Mn than those of Zn (−0.76 V vs. SHE; 820 mAh g^−1^). In our case, although the Mn||MnVO cells display lower specific capacity (based on the mass of MnVO) than the case of Zn||MnVO cells, they can still deliver higher specific energy due to the higher discharge voltage (Supplementary Fig. 31). In addition, with the rational design of cathode architectures, aqueous MIBs could deliver stable cycling performance and excellent rate capability. More importantly, the rich valence states of manganese (Mn^0^, Mn^2+^, Mn^3+^, Mn^4+^, and Mn^7+^) would provide great opportunities for the exploration of various manganese-based battery systems^20^.

In spite of the advantages mentioned above, there is still much work to do for MIBs to promote their practical applications. The main challenge is to improve the reversibility of Mn plating/stripping in the charge/discharge process. Mn^2+^ ions could strip from Mn/C anodes during discharging (Supplementary Fig. 32), leading to high cell discharge voltages of aqueous MIBs. However, in the reversible charging process, hydrogen evolution would occur at anode since the redox potential of hydrogen evolution is higher than that of Mn^2+^/Mn, resulting in poor reversibility of the Mn plating/stripping process. During the hydrogen evolution, a large amount of OH^−^ ions yields. These OH^−^ ions further combine with Mn^2+^ ions to form Mn(OH)_2_ nanosheets on the surface of the anode (Supplementary Fig. 33). The Mn(OH)_2_ layer at the anode will impede ion transportation, degrading cycling stability (Supplementary Fig. 34 and Supplementary Note 3). Since such a side reaction occurs, the charge/discharge capacity degrades gradually after 100 charge/discharge cycles in the case of Mn||MnVO cells. In Mn||4-Cl-BQ cells, the long cycling performance was also limited. To further promote the long cycling behavior, electrolyte or anode optimizing has to be considered. In the case of electrolytes, “water in salt” or molecular crowded electrolytes could be employed to broaden the voltage window of aqueous electrolytes and suppress the hydrogen evolution^1,58^. Besides, the electrodeposition efficiency of Mn metal is also important for long cycling performance. Some strategies, such as lattice matching, alloying of Mn metal, and the formation of a favorable solid electrolyte interphase on the anode surface, could be developed to improve the reversibility of Mn plating/striping^59^. In addition, the design of active materials is also crucial to further improve the electrochemical performance of aqueous MIBs. Moreover, the exploration of cathode materials is accompanied by the emergence of other energy storage mechanisms.

## Discussion

The reversible Mn^2+^ ion intercalation process was achieved in aqueous battery systems, in which two kinds of cathodes, an inorganic material, and organic material, have been proved to possess the Mn^2+^ ion storage ability. The layered MnVO cathode displays the reversible Mn^2+^ ion insertion/extraction mechanism with fast kinetics and stable cycling performance during the discharge/charge process. The Mn||MnVO cell can be fully charged to 1.9 V at around 20 s (10.0 A g^−1^, 64.8 Wh kg^−1^), suggesting the fast-charging capabilities. Another organic cathode of 4-Cl-BQ exhibits an Mn^2+^ ion coordination/uncoordination with the transformation between C=O and C–O^−^ groups. The DFT calculations suggest that one Mn^2+^ ion will coordinate with two C–O^−^ groups of adjacent 4-Cl-BQ molecules. The resultant Mn||4-Cl-BQ cell shows a stable discharge voltage at ~1.37 V. This work will broaden the battery research to Mn^2+^ ions as charge carriers and also help to improve the electrochemical performance of aqueous batteries.

## Methods

### Materials

Vanadium pentoxide (V_2_O_5_, 99%), manganese sulfate monohydrate (MnSO_4_·H_2_O, 99%), manganese nitrate tetrahydrate (Mn(NO_3_)_2_·4H_2_O, 98%) and hydrogen peroxide (H_2_O_2_, 30%) were purchased from Aladdin. Manganese chloride tetrahydrate (MnCl_2_·4H_2_O, 99.9%) and N-methyl-2-pyrrolidone (NMP, 99.5%) were purchased from Macklin. Super P, polyvinylidene fluoride (PVDF) and trichloromethane (CHCl_3_, 99%) were from Sinopharm Chemical Reagent Co., Ltd. Ttetrachloro-1,4-benzoquinone (4-Cl-BQ, 98%) was from Meryer Chemical Technology Co., Ltd. Glass fiber (Grade GF/A with a thickness of 260 µm) was purchased from Whatman. Manganese bis(trifluoromethanesulfonate) (Mn(CF_3_SO_3_)_2_) and zinc bis(trifluoromethanesulfonate) (Zn(CF_3_SO_3_)_2_, 97.5%) were from Sigma-Aldrich and J&K Scientific Ltd., respectively. They were purified via filtration by syringe before using. A measure of 0.353 g of Mn(CF_3_SO_3_)_2_ was added into 1 mL deionized water under magnetic stirring. The Mn(CF_3_SO_3_)_2_ aqueous solution was transferred into a syringe filter and then purified by the aqueous syringe-driven filter to obtain Mn(CF_3_SO_3_)_2_ electrolyte (1 M). The purification of Zn(CF_3_SO_3_)_2_ electrolyte (1 M) follows the same steps. Mn particles (99.8%, Supplementary Fig. 35a–c) and Zn foil (99.9%) were purchased from HeBei Xindun of China and Alfa Aesar, respectively. They were used directly without any treatment. Stainless steel (>99%, ~56 µm) and carbon felts (~330 µm) were purchased from Tianhong Wangye and Taiwan Tanneng of China, respectively.

### Electrode preparation

A 2 mmol of commercial V_2_O_5_ powder was dissolved into 50 mL of deionized water with 2 mL of H_2_O_2_. A 0.2 mmol of MnSO_4_ was dissolved into 30 mL of deionized water. These two solutions were transferred to a 100 mL Teflon-lined stainless steel autoclave and then heated at 120 °C for 5 h. After the reaction, the suspension was washed with deionized water several times and finally, the MnVO nanosheets were obtained by freeze-drying (−80 °C). MnVO nanosheets, super P, and PVDF were mixed with a weight ratio of 7:2:1 by NMP. The slurry was grinded manually in mortar for 1 h and then coated on stainless steel (0.785 cm^2^). After drying at 80 °C for 12 h under vacuum, MnVO cathode was achieved. The mass loading of MnVO in the cathode (~80 µm, which is the thickness of stainless steel with the coating) was ~1.0 mg cm^−2^ ± 0.5 mg cm^−2^.

1 mL Vitamin C (1 mol L^−1^) and 20 mL GO dispersion (1 mg mL^−1^) were mixed and then sealed in a 50 mL Teflon lined stainless steel autoclave. The mixed solution was maintained at 95 °C. After 10 h, rGO hydrogel was achieved. It was washed, cut, and then freeze-dried to obtain rGO foams (750 µm ± 250 µm). Commercial 4-Cl-BQ was dissolved in CHCl_3_ to obtain the yellow solution with a concentration of 2 mg mL^−1^. Then, single-channel adjustable pipettes were employed to drip the resultant solution with a specific volume of ~500 μL/cm^−2^ into rGO foams. After drying, 4-Cl-BQ@rGO electrodes (4-Cl-BQ, ~40%) were obtained. The mass loading of 4-Cl-BQ in electrodes was ~1.0 mg cm^−2^ ± 0.5 mg cm^−2^.

Commercial Mn particles, super P, and PVDF were mixed with a weight ratio of 8:1:1 by NMP. The slurry was grinded manually in mortar for 1 h and then coated onto carbon felts (0.785 cm^2^). The carbon felts were then dried at 80 °C for 12 h under vacuum to obtain the Mn/C composite anodes (Supplementary Fig. 35d). The thickness of the carbon felt with the Mn/C coating was ~500 µm and the mass loading of Mn metal was >30 mg cm^−2^.

### Electrochemical measurement

All the electrochemical measurements were carried out using CR2032 coin cells, in which the areas of electrodes and glass fiber separators were 0.785 and 2.01 cm^2^, respectively. The amount of electrolyte in a cell was 200 μL. Mn metal cells were assembled with MnVO or 4-Cl-BQ@rGO cathode, Mn/C anode, and 1 M Mn-containing aqueous electrolyte. Zn||MnVO cells were assembled with MnVO cathode, Zn foil anode, and 1 M Zn(CF_3_SO_3_)_2_ electrolyte. Mn/C||Mn/C symmetric cells were tested with identical Mn/C electrodes and 1 M Mn(CF_3_SO_3_)_2_ electrolyte.

CV (scan rate: 1.0–3.0 mV s^−1^) and linear sweep voltammetry (LSV) (scan rate: 10.0 mV s^−1^) measurements were carried out with electrochemical workstations (CHI 660E). GCD (specific current: 0.05–10.0 A g^−1^) and GITT measurements (specific current: 0.2 A g^−1^) were performed on a cell test system (LAND, CT2001A). The voltage windows of Mn||MnVO, Zn||MnVO, and Mn||4-Cl-BQ cells were 1.0–1.9, 0.7–1.6, and 0.8–1.8 V, respectively. Electrochemical impedance spectroscopy measurements of the Mn/MnVO batteries were examined in a two-electrode cell configuration, in which Mn/C, MnVO, and 1 M Mn(CF_3_SO_3_)_2_ or MnSO_4_ served as an anode, cathode, and electrolyte, respectively. The tests were performed in a frequency range of 100 kHz to 10 mHz (Zennium E, Zahner Elektrik GmbH & Co. KG) by applying a disturbance amplitude of 5 mV. The number of data points was 59. The quasi-stationary potential was maintained during the measurement. All the electrochemical energy storage tests were carried out at around 25 °C in an environmental chamber.

The electrodeposition of Mn metal on stainless steel was carried out by LSV via two-electrode cell configuration, where stainless steel, Mn/C, and 1 M Mn-contained aqueous solution served as working electrode, a counter electrode, and electrolyte, respectively. The LSV curves were measured from the open-circuit voltage to −1.5 V with a scan rate of 10 mV s^−1^. The deposition in different aqueous electrolytes (MnSO_4_ or Mn(CF_3_SO_3_)_2_) was performed following the same steps.

The specific energy (*E*, Wh kg^−1^) and power (*P*, W kg^−1^) of batteries based on active materials were calculated as follows^49^:

4 $$E=\frac{\int {IV}\left(t\right){dt}}{3.6m}$$

5 $$P=\frac{3600E}{t}$$

in which *I* (A) and *V* (V) are the current and voltage of the cell, *t* (s) is the discharge time, and *m* (g) is the weight of active material. The voltage of the cell was the average discharge voltage, which was at the half of the discharge capacity in discharging process. In calculating the energy and power densities in Fig. 6a, the mass of devices is regarded as five times of the active materials in cathodes.

### Characterization

The morphologies of MnVO nanosheets, MnVO cathodes, commercial Mn particles, Mn/C composite anodes, rGO foams, commercial 4-Cl-BQ sheets, and 4-Cl-BQ@rGO composites were characterized by field-emission scanning electron microscopy (JEOLJSM-7500F) equipped with EDX for elemental analysis. The microstructure was characterized via TEM (Talos F200X G2) equipped with EDX for elemental analysis. XRD patterns were obtained from Rigaku Ultima IV with Cu Kα radiation. FTIR spectra were collected on Bruker Tensor II. Raman spectra were obtained from a confocal Raman microscope (DXR, Thermo Fisher Scientific) with a 532 nm excitation from an argon-ion laser. XPS tests were performed through PerkinElmer PHI 1600 ESCA. Thermogravimetric analysis (Netzsch STA 449 F3 Jupiter analyzer) was carried out from room temperature to 800 °C (Ar flow, 10 °C min^−1^). ICP-AES tests were carried out from PerkinElmer Optima 8300. The contact angles of electrolytes were obtained on Physics OCA 25. For the ex situ measurements at different charge/discharge states, the electrodes were washed with deionized water more than 5 times and then dried in the atmosphere for further characterization without any specific transport holder.

The amount of the inserted/extracted Mn^2+^ ions in MnVO was determined by ICP-AES, which was obtained according to the formula^36^:

6 $$I=a{C}^{b}$$

where *I* and *C* are the intensity of characteristic spectra and the concentration of metal elements, respectively; *a* and *b* are adjustable parameters. The standard curves of Mn and V were obtained via measuring standard solutions with different concentrations of Mn and V. The MnVO electrodes were first washed with deionized water 5 times and then dissolved in 5 mL 0.5 M H_2_SO_4_. After purifying via removing super P and PVDF, the solution was achieved and then diluted to 500 mL for further characterization. The molar ratios of Mn and V could be quantified according to the above formula.

### Computational methods

The spin-polarized first-principles calculations based on the DFT were performed using the Vienna Ab-initio Simulation Package with projector-augmented-wave pseudopotentials. The generalized gradient approximation of Perdew–Burke–Ernzerhof was employed for the exchange-correlation function. Cutoff energy of 500 eV was selected as the plane-wave basis to ensure the precision of the calculations using a 2 × 3 × 2 Monkhorst–Pack reciprocal space grid of k-points for a single unit cell. The relaxation of the electronic degrees of freedom was assumed to be converged when the total energy change between the two electronic optimization steps was smaller than 1 × 10^−6^ eV. Geometry relaxation was carried out before studying the structural properties and total energy until all forces on each atom were below 0.02 eV Å^−1^.

## Supplementary information

Supplementary Information

## Data Availability

The authors declare that all the relevant data are available within the paper and its Supplementary Information file or from the corresponding author upon reasonable request.

## References

[1] Suo L (2015). “Water-in-salt” electrolyte enables high-voltage aqueous lithium-ion chemistries. Science.

[2] Huang Z (2021). Manipulating anion intercalation enables a high-voltage aqueous dual ion battery. Nat. Commun..

[3] Yang C (2019). Aqueous Li-ion battery enabled by halogen conversion-intercalation chemistry in graphite. Nature.

[4] Yamada Y (2016). Hydrate-melt electrolytes for high-energy-density aqueous batteries. Nat. Energy.

[5] Yang C (2017). 4.0 V aqueous Li-ion batteries. Joule.

[6] Wu X (2019). A rechargeable battery with an iron metal anode. Adv. Funct. Mater..

[7] Xu C, Li B, Du H, Kang F (2012). Energetic zinc ion chemistry: the rechargeable zinc ion battery. Angew. Chem. Int. Ed..

[8] Yan C (2020). Architecting a stable high-energy aqueous Al-ion battery. J. Am. Chem. Soc..

[9] Wang S (2021). Non-metal ion co-insertion chemistry in aqueous Zn/MnO_2_ batteries. Angew. Chem. Int. Ed..

[10] Tang X (2021). A universal strategy towards high-energy aqueous multivalent-ion batteries. Nat. Commun..

[11] Liu Z (2019). Voltage issue of aqueous rechargeable metal-ion batteries. Chem. Soc. Rev..

[12] Qiu H (2019). Zinc anode-compatible in-situ solid electrolyte interphase via cation solvation modulation. Nat. Commun..

[13] Wu C (2019). Electrochemically activated spinel manganese oxide for rechargeable aqueous aluminum battery. Nat. Commun..

[14] Yang H (2019). Progress in rechargeable aqueous zinc-and aluminum-ion battery electrodes: challenges and outlook. Angew. Chem. Int. Ed..

[15] Kravchyk KV, Kovalenko MV (2020). The pitfalls in nonaqueous electrochemistry of Al-ion and Al dual-ion batteries. Adv. Energy Mater..

[16] Shen X (2021). Ultra-fast charging in aluminum-ion batteries: electric double layers on active anode. Nat. Commun..

[17] Faegh E, Ng B, Hayman D, Mustain WE (2020). Practical assessment of the performance of aluminium battery technologies. Nat. Energy.

[18] Tu J (2021). Nonaqueous rechargeable aluminum batteries: progresses, challenges, and perspectives. Chem. Rev..

[19] Chao D (2020). Roadmap for advanced aqueous batteries: from design of materials to applications. Sci. Adv..

[20] Chen W (2018). A manganese-hydrogen battery with potential for grid-scale energy storage. Nat. Energy.

[21] Zhang K (2015). Nanostructured Mn-based oxides for electrochemical energy storage and conversion. Chem. Soc. Rev..

[22] Díaz-Arista P (2006). EQCM study of the electrodeposition of manganese in the presence of ammonium thiocyanate in chloride-based acidic solutions. Electrochim. Acta.

[23] Dong H (2020). High-power Mg batteries enabled by heterogeneous enolization redox chemistry and weakly coordinating electrolytes. Nat. Energy.

[24] Huang S, Zhu J, Tian J, Niu Z (2019). Recent progress in the electrolytes of aqueous zinc-ion batteries. Chem. Eur. J..

[25] Zhang N (2016). Cation-deficient spinel ZnMn_2_O_4_ cathode in Zn(CF_3_SO_3_)_2_ electrolyte for rechargeable aqueous Zn-ion battery. J. Am. Chem. Soc..

[26] Wan F (2018). An aqueous rechargeable zinc-organic battery with hybrid mechanism. Adv. Funct. Mater..

[27] Liang G, Mo F, Ji X, Zhi C (2020). Non-metallic charge carriers for aqueous batteries. Nat. Rev. Mater..

[28] Xu Y (2019). Vanadium oxide pillared by interlayer Mg^2+^ ions and water as ultralong-life cathodes for magnesium-ion batteries. Chemistry.

[29] Xu X (2019). Bilayered Mg_0.25_V_2_O_5_·H_2_O as a stable cathode for rechargeable Ca-ion batteries. ACS Energy Lett..

[30] Kundu D, Adams BD, Duffort V, Vajargah SH, Nazar LF (2016). A high-capacity and long-life aqueous rechargeable zinc battery using a metal oxide intercalation cathode. Nat. Energy.

[31] Yan M (2018). Water-lubricated intercalation in V_2_O_5_·nH_2_O for high-capacity and high-rate aqueous rechargeable zinc batteries. Adv. Mater..

[32] Lu Y, Chen J (2020). Prospects of organic electrode materials for practical lithium batteries. Nat. Rev. Chem..

[33] Tie Z, Niu Z (2020). Design strategies for high-performance aqueous Zn/organic batteries. Angew. Chem. Int. Ed..

[34] Kim D (2018). Rechargeable aluminium organic batteries. Nat. Energy.

[35] Zhao Q (2018). High-capacity aqueous zinc batteries using sustainable quinone electrodes. Sci. Adv..

[36] Wan F (2018). Aqueous rechargeable zinc/sodium vanadate batteries with enhanced performance from simultaneous insertion of dual carriers. Nat. Commun..

[37] Zhao Q (2019). Proton intercalation/de-intercalation dynamics in vanadium oxides for aqueous aluminum electrochemical cells. Angew. Chem. Int. Ed..

[38] Wang L, Huang K, Chen J, Zheng J, Maia Y (2019). Ultralong cycle stability of aqueous zinc-ion batteries with zinc vanadium oxide cathodes. Sci. Adv..

[39] He P (2017). High-performance aqueous zinc-ion battery based on layered H_2_V_3_O_8_ nanowire cathode. Small.

[40] Zhang G (2021). Rich alkali ions preintercalated vanadium oxides for durable and fast zinc-ion storage. ACS Energy Lett..

[41] Dong S (2019). Ultra-fast NH_4_^+^ storage: strong H bonding between NH_4_^+^ and bi-layered V_2_O_5_. Chemistry.

[42] Kundu D (2018). Organic cathode for aqueous Zn-ion batteries: taming a unique phase evolution toward stable electrochemical cycling. Chem. Mater..

[43] Tie Z, Liu L, Deng S, Zhao D, Niu Z (2020). Proton insertion chemistry of Zn/organic battery. Angew. Chem. Int. Ed..

[44] Guo Z (2018). Environment-friendly and flexible aqueous zinc battery using an organic cathode. Angew. Chem. Int. Ed..

[45] Guo Z (2020). An organic/inorganic electrode-based hydronium-ion battery. Nat. Commun..

[46] Emanuelsson R, Sterby M, Stromme M, Sjodin M (2017). An all-organic proton battery. J. Am. Chem. Soc..

[47] Liang Y (2017). Universal quinone electrodes for long cycle life aqueous rechargeable batteries. Nat. Mater..

[48] Jiang L (2019). Building aqueous K-ion batteries for energy storage. Nat. Energy.

[49] Han C, Li H, Li Y, Zhu J, Zhi C (2021). Proton-assisted calcium-ion storage in aromatic organic molecular crystal with coplanar stacked structure. Nat. Commun..

[50] Wang F (2017). High-voltage aqueous magnesium ion batteries. ACS Cent. Sci..

[51] Li Y (2021). High-energy-density quinone-based electrodes with [Al(OTF)]^2+^ storage mechanism for rechargeable aqueous aluminum batteries. Adv. Funct. Mater..

[52] Wang Y (2020). Binding zinc ions by carboxyl groups from adjacent molecules toward long-life aqueous zinc-organic batteries. Adv. Mater..

[53] Li Y (2019). Rechargeable aqueous polymer-air batteries based on polyanthraquinone anode. Chemistry.

[54] Zhang N (2019). Hydrated layered vanadium oxide as a highly reversible cathode for rechargeable aqueous zinc batteries. Adv. Funct. Mater..

[55] Bi S (2020). Free-standing three-dimensional carbon nanotubes/amorphous MnO_2_ cathodes for aqueous zinc-ion batteries with superior rate performance. Mater. Today Energy.

[56] Yue F (2021). Ultralow temperature aqueous battery with proton chemistry. Angew. Chem. Int. Ed..

[57] Wei Z (2019). Reversible intercalation of methyl viologen as a dicationic charge carrier in aqueous batteries. Nat. Commun..

[58] Xie Z, Liang Z, Lu Y (2020). Molecular crowding electrolytes for high-voltage aqueous batteries. Nat. Mater..

[59] Zheng J (2019). Reversible epitaxial electrodeposition of metals in battery anodes. Science.

[60] Jia X, Liu C, Neale ZG, Yang J, Cao G (2020). Active materials for aqueous zinc ion batteries: synthesis, crystal structure, morphology, and electrochemistry. Chem. Rev..

