# Supplementary material for: A rechargeable aqueous manganese-ion battery based on intercalation chemistry
Source: Nat Commun. 2021 Nov 30;12:6991. doi: 10.1038/s41467-021-27313-5 (PMC8632892; doi:10.1038/s41467-021-27313-5)
Supplement: Supplementary file 1 — Supplementary Information [file 41467_2021_27313_MOESM1_ESM.pdf]

Supplementary Information for

A rechargeable aqueous manganese-ion battery based on intercalation chemistry

*Songshan Bi[1], Shuai Wang[1], Fang Yue[1], Zhiwei Tie[1] and Zhiqiang Niu[1]**

[1]Key Laboratory of Advanced Energy Materials Chemistry (Ministry of Education), College of Chemistry, Nankai University, Tianjin, 300071, P. R. China.

*The corresponding authors, Email address: zqniu@nankai.edu.cn,

Supplementary Figures

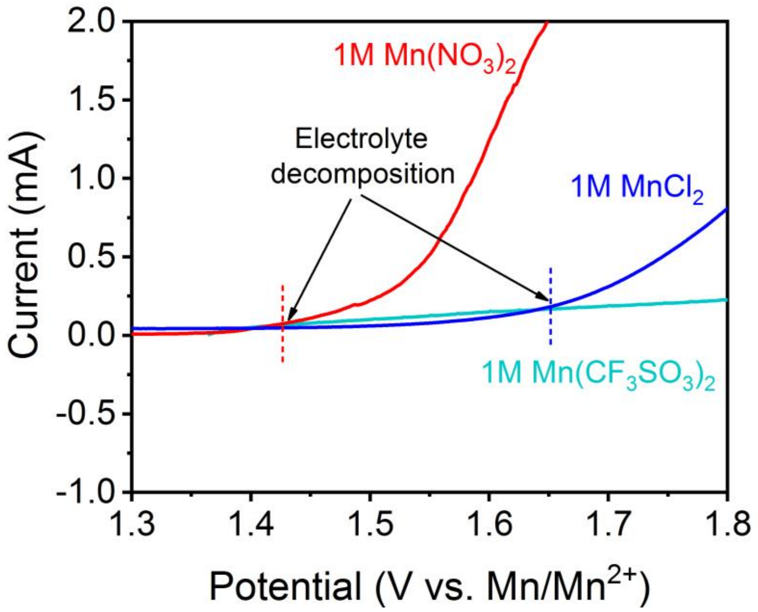

Supplementary Figure 1. LSV curves of various aqueous Mn-contained electrolytes.

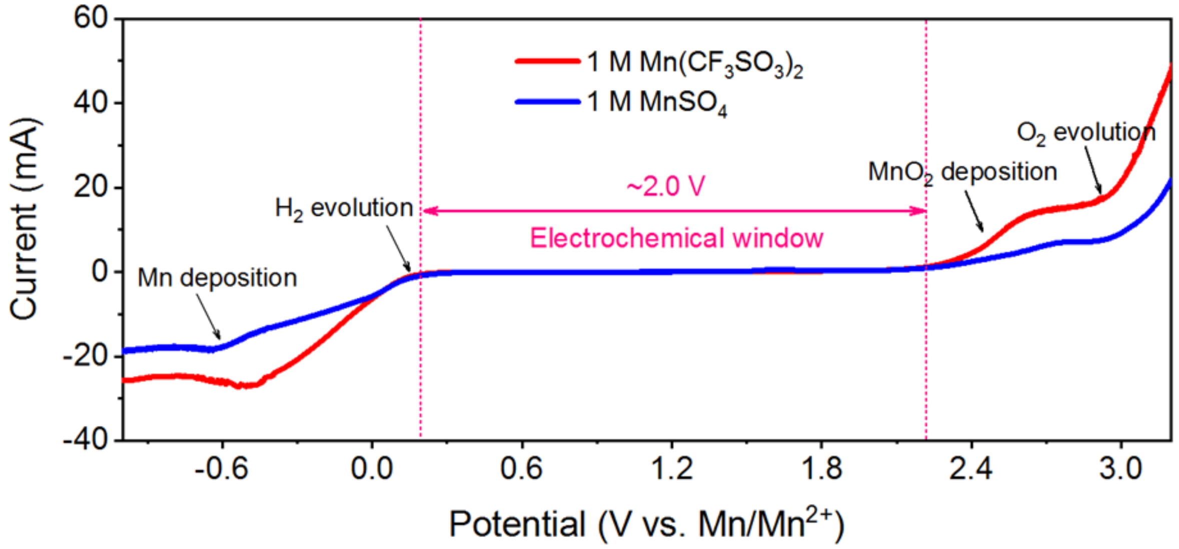

Supplementary Figure 2. Electrochemical window of $MnSO_4$ and $Mn(CF_3SO_3)_2$ electrolytes at 10.0 mV s^{-1}.

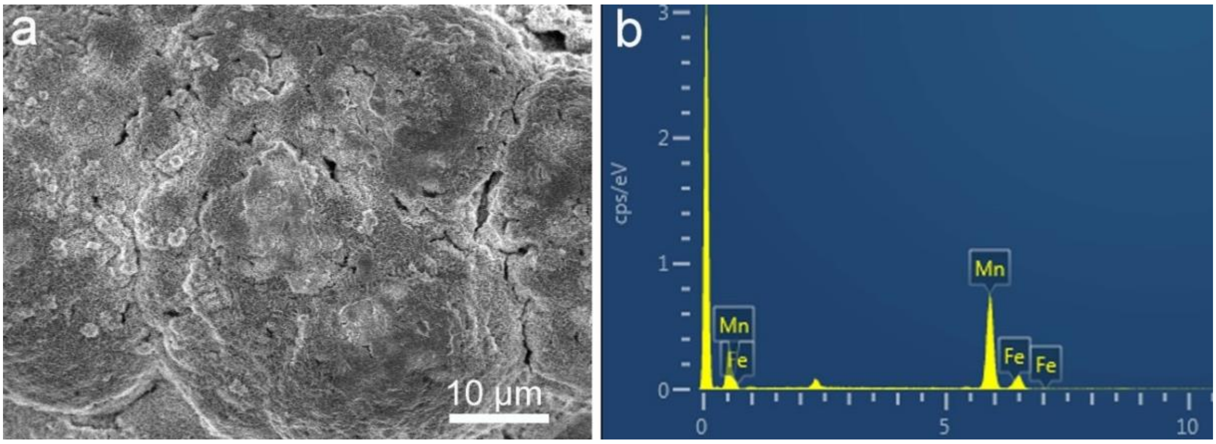

Supplementary Figure 3. a SEM image and **b** EDX result of Mn, which was electrodeposited on stainless steel in $Mn(CF_3SO_3)_2$ electrolyte.

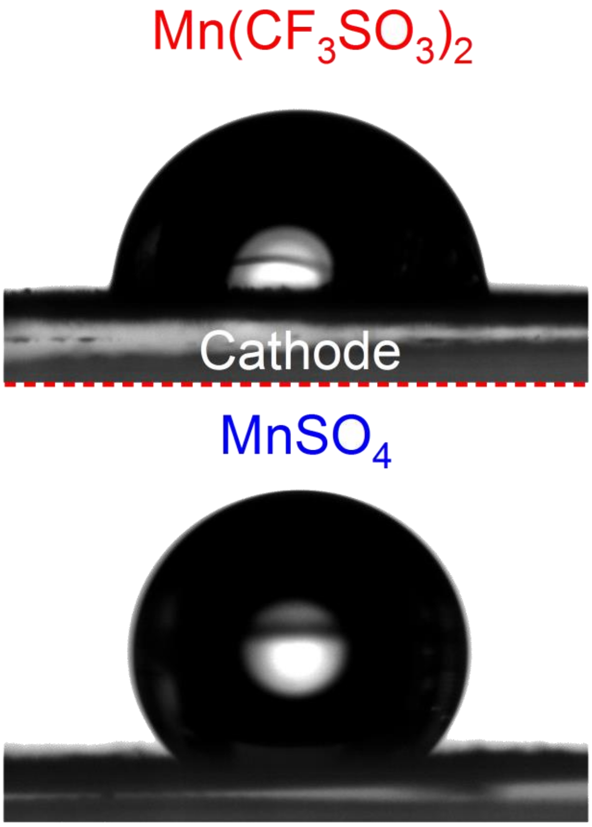

Supplementary Figure 4. Contact angles of 1.0 M $MnSO_4$ and $Mn(CF_3SO_3)_2$ electrolytes with the MnVO cathode.

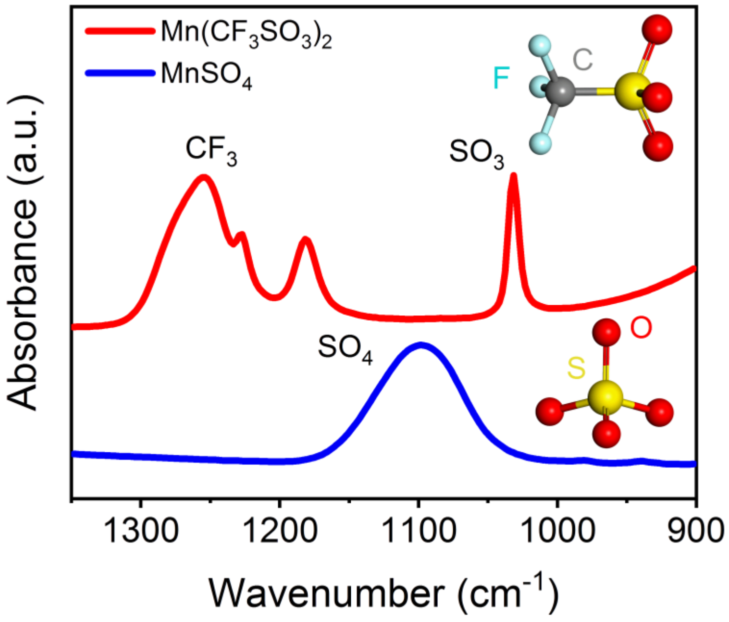

Supplementary Figure 5. FTIR spectra of $MnSO_4$ and $Mn(CF_3SO_3)_2$ electrolytes. Inserts are the typical structures of SO_4^{2-} and $CF_3SO_3^-$.

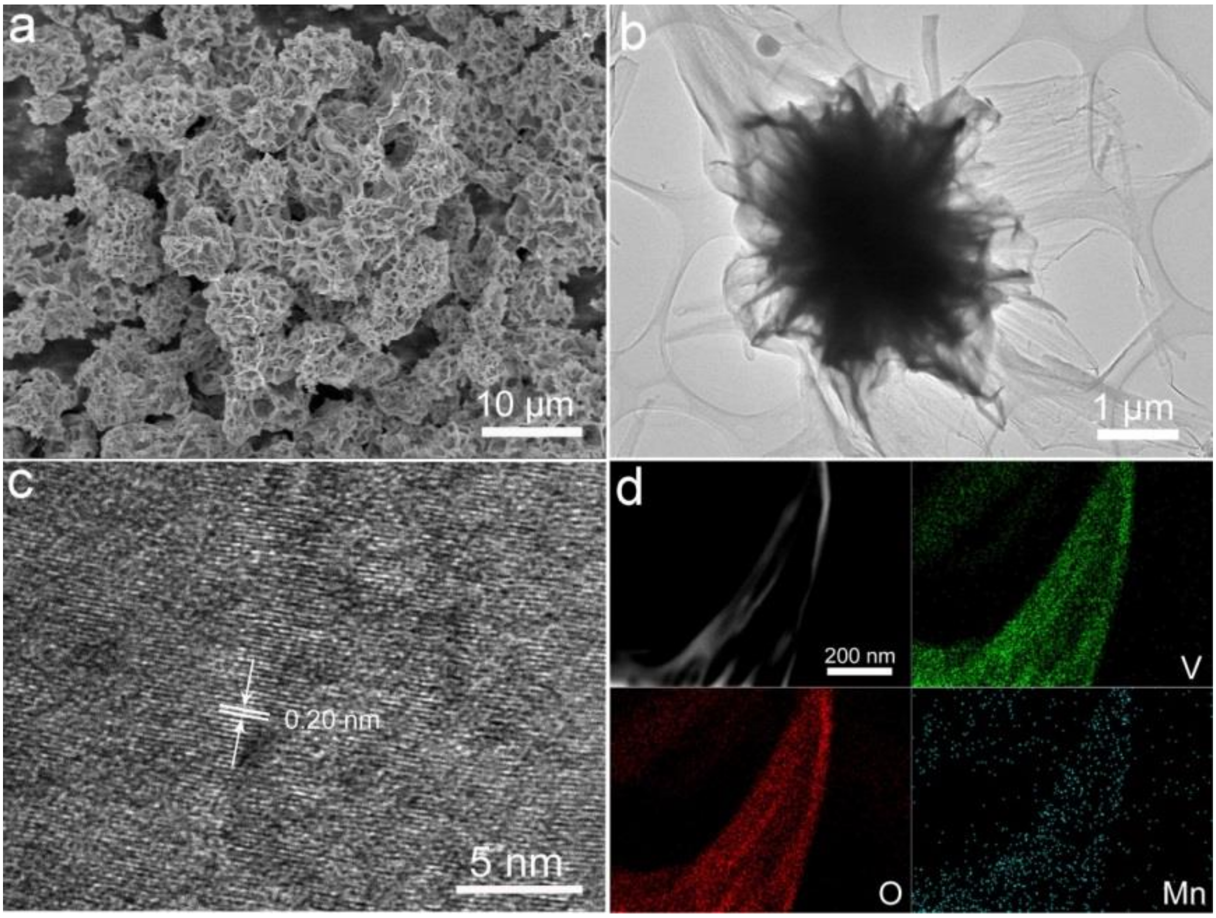

Supplementary Figure 6. a SEM, **b** TEM, **c** high-resolution TEM images and **d** element mapping of MnVO.

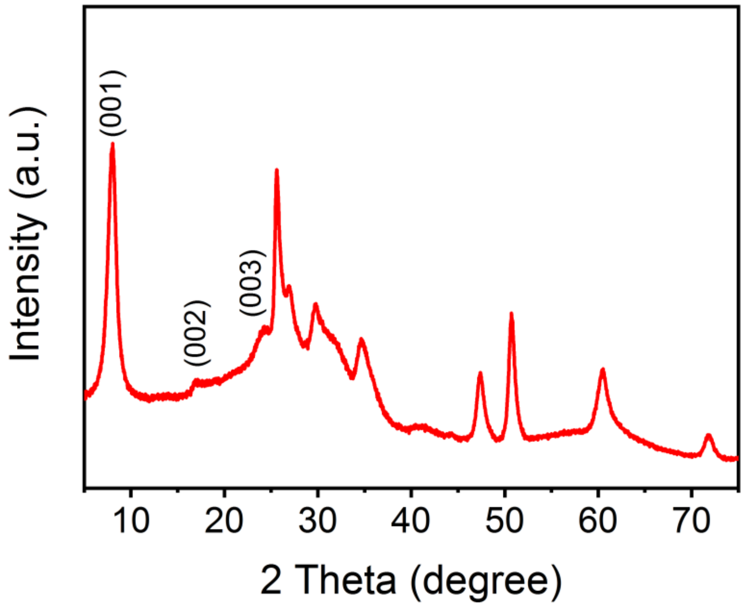

Supplementary Figure 7. XRD pattern of MnVO.

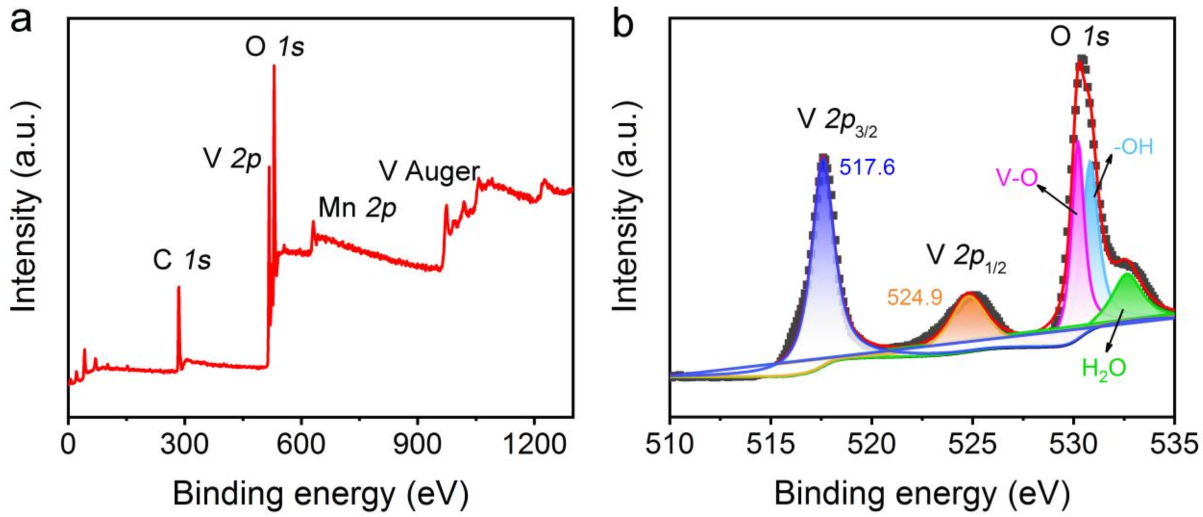

Supplementary Figure 8. XPS spectra of **a** full spectrum, **b** V *2p* and O *1s* of MnVO.

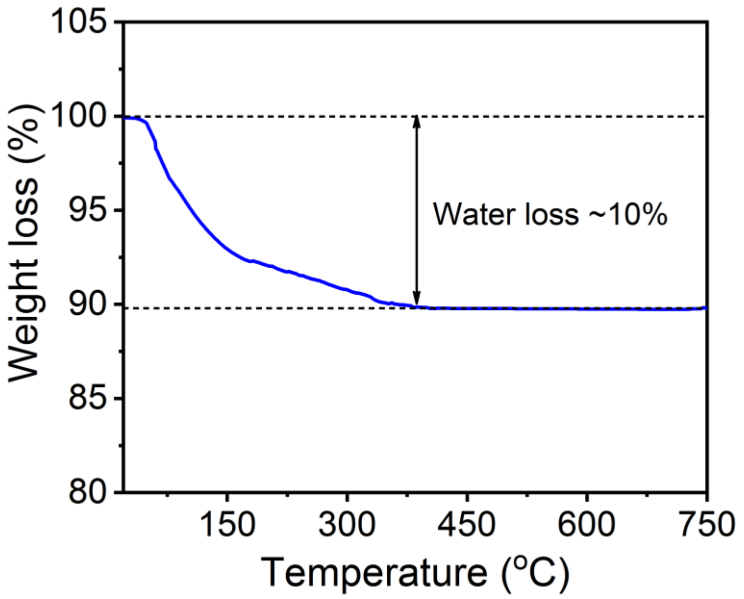

Supplementary Figure 9. TGA of MnVO.

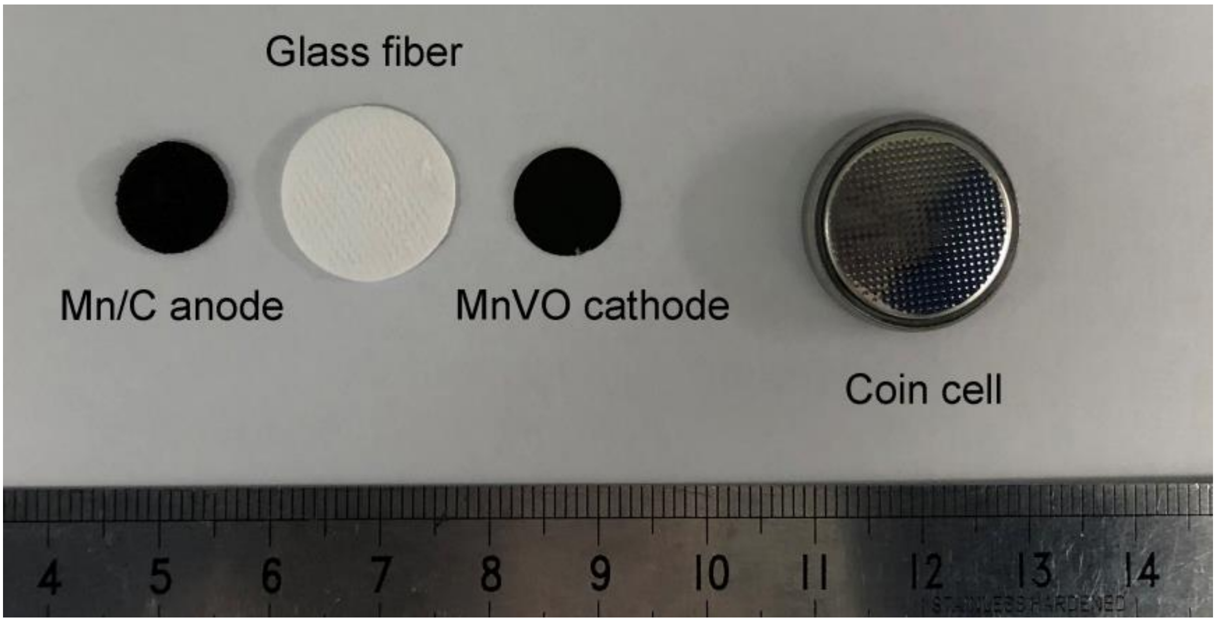

Supplementary Figure 10. Optical image of the anode, separator, cathode and the assembled coin cell.

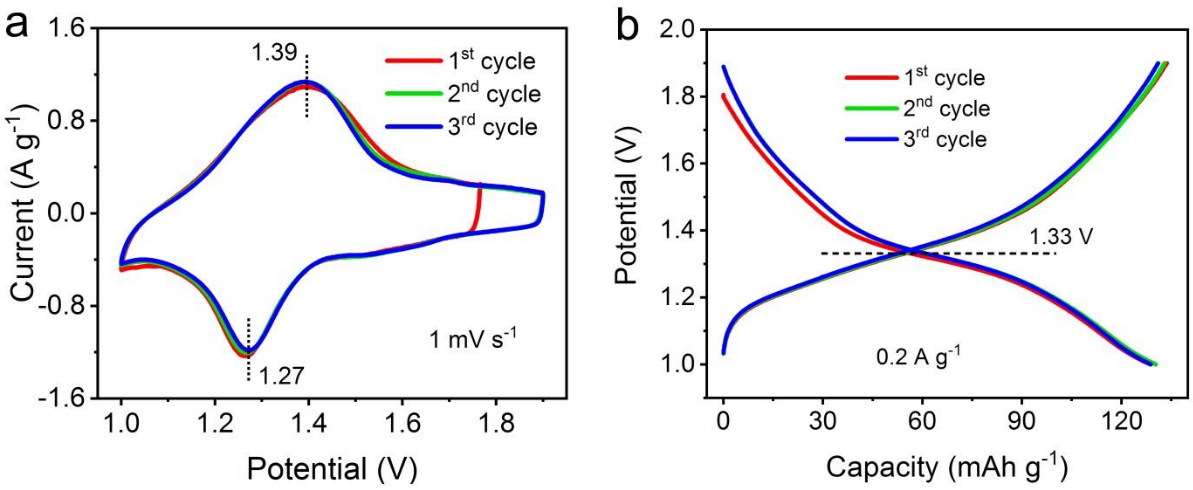

Supplementary Figure 11. a CV curves at 1.0 mV s^{-1} and **b** GCD curves at 0.2 A g^{-1} of Mn||MnVO cells.

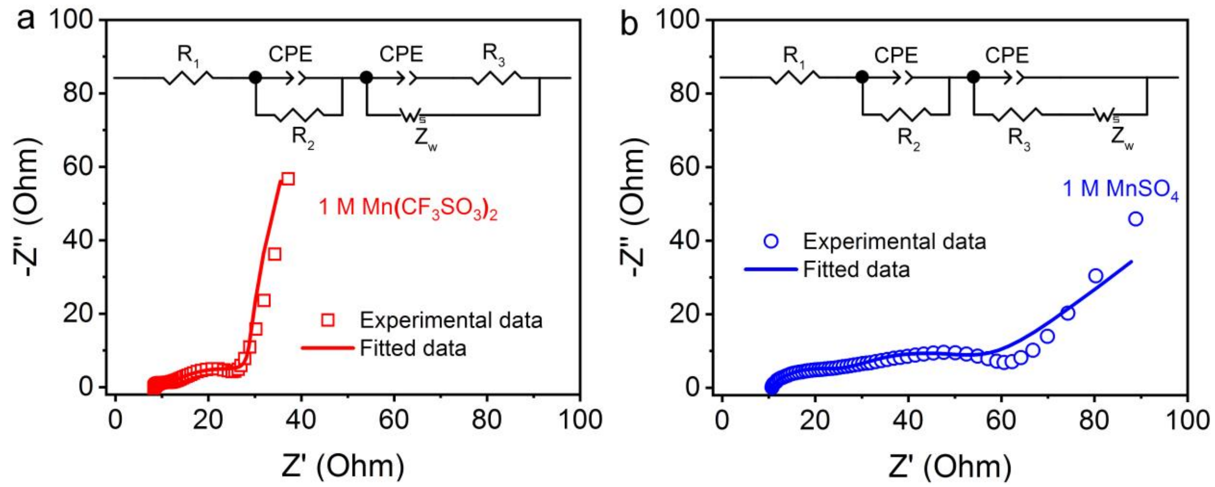

Supplementary Figure 12. The fitted curves and the equivalent circuits of the Nyquist plots of Mn||MnVO cells in **a** $Mn(CF_3SO_3)_2$ and **b** $MnSO_4$ electrolytes, where R_1, R_2, R_3, CPE, and Z_w represent series resistance, interface resistance between electrolyte and electrode, charge-transfer resistance, constant-phase element, and Warburg diffusion process.

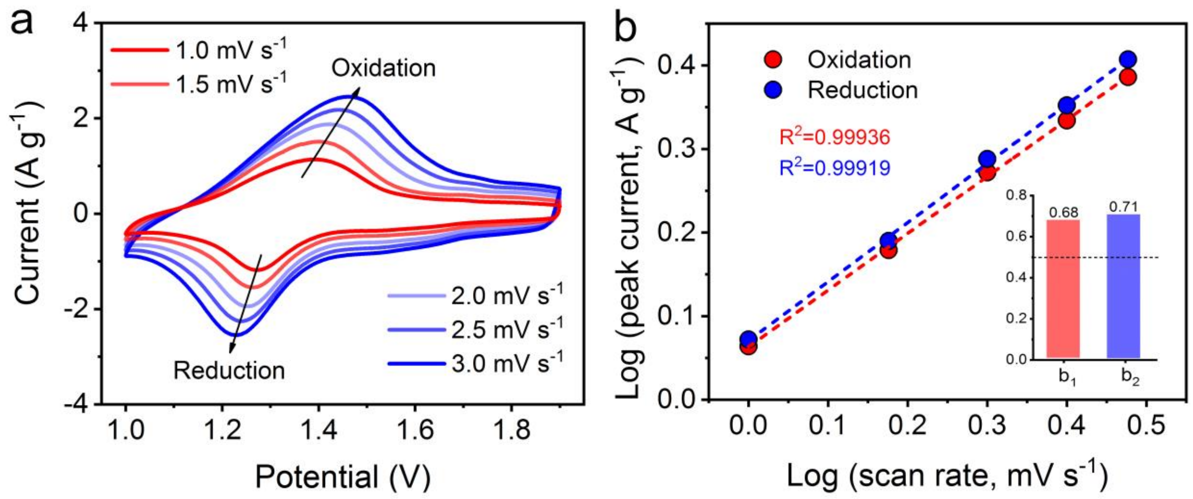

Supplementary Figure 13. a CV curves of Mn||MnVO cell at different scan rates. **b** The relationship between peak currents and scan rates in CV curves. Insert in **b** is the calculated *b* values of the oxidation/reduction peaks.

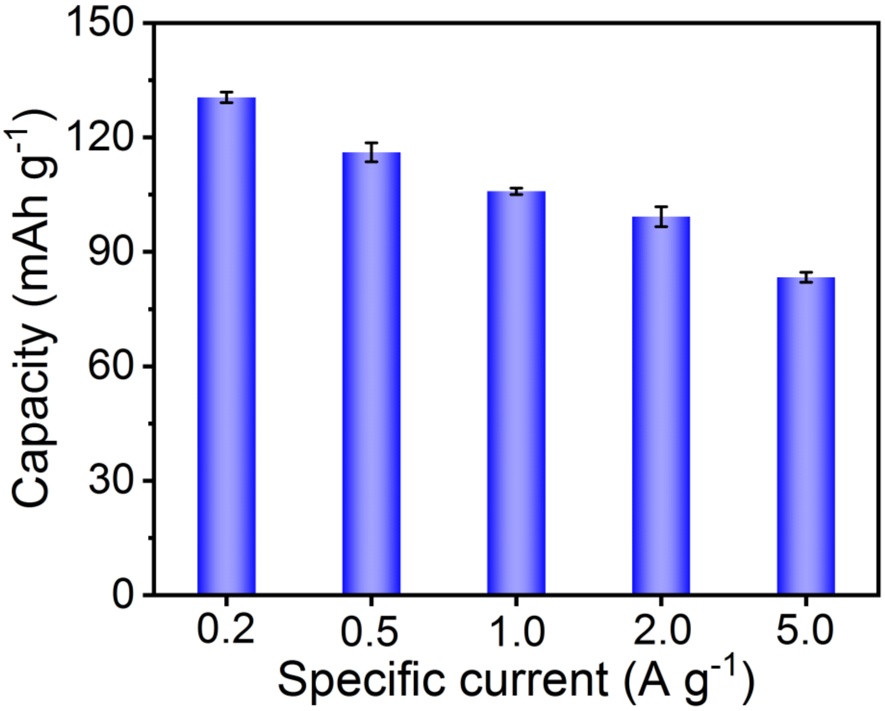

Supplementary Figure 14. Capacity of the Mn||MnVO cells at different specific currents. Error bars indicate the standard deviations of capacities from three different cells.

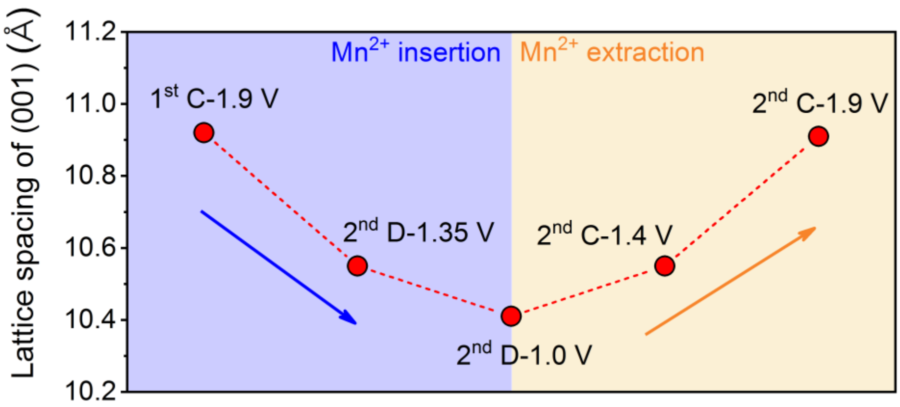

Supplementary Figure 15. Lattice spacing evolution of the (001) plane of MnVO at different charge/discharge states.

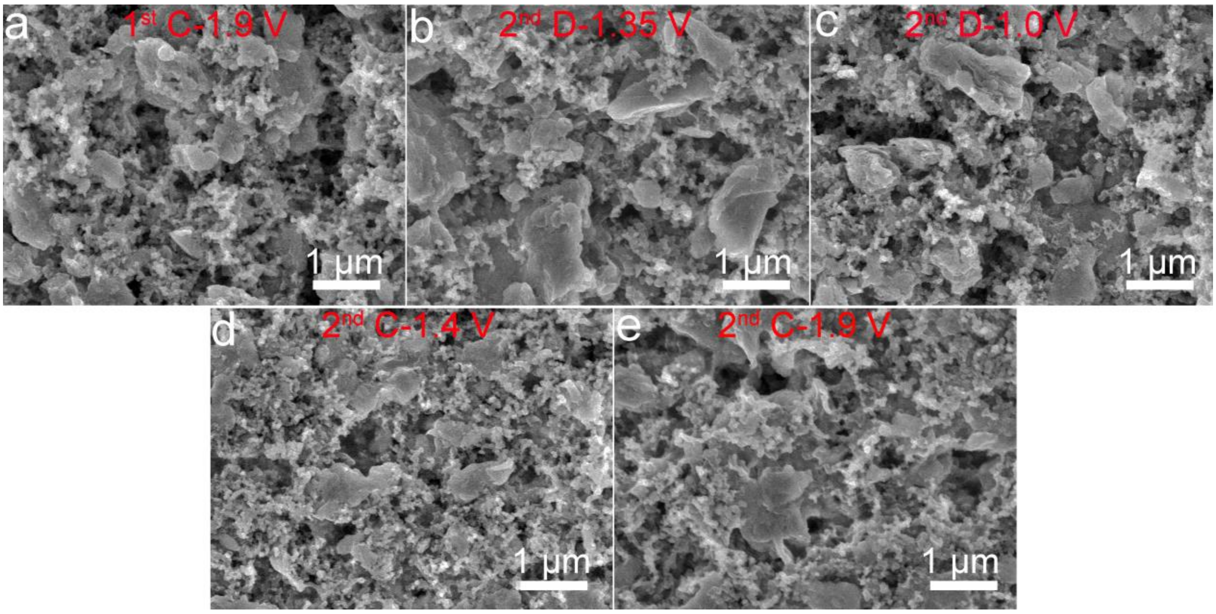

Supplementary Figure 16. SEM images of MnVO cathodes at different charge/discharge states.

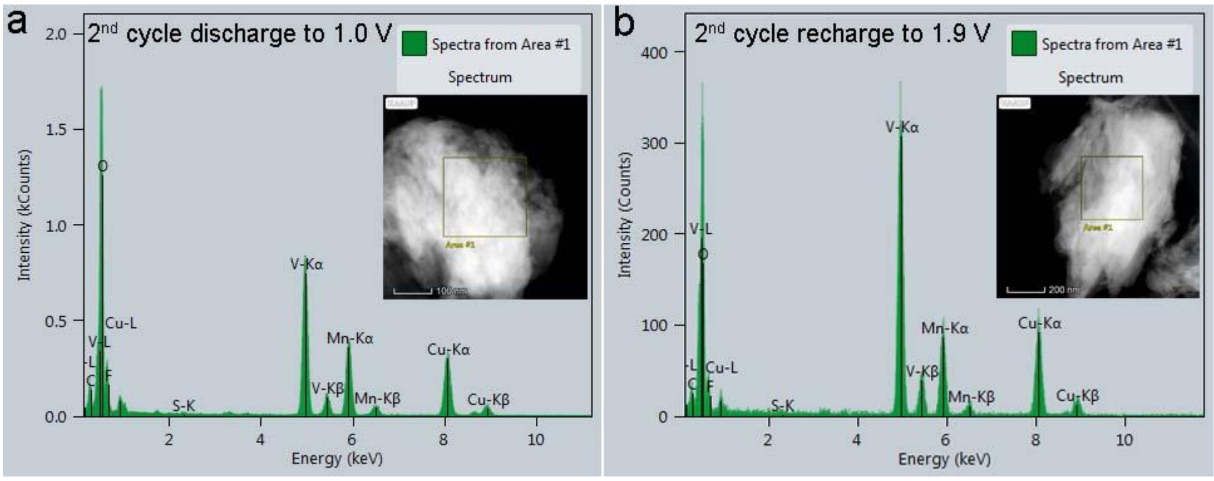

Supplementary Figure 17. EDX results of MnVO cathodes at **a** discharged and **b** recharged states.

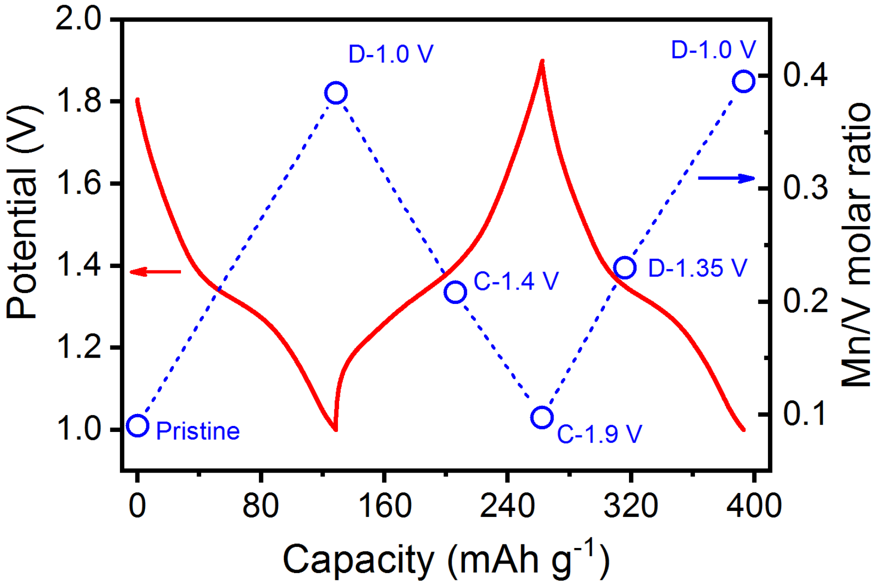

Supplementary Figure 18. ICP-AES study of MnVO cathodes at different charge/discharge states.

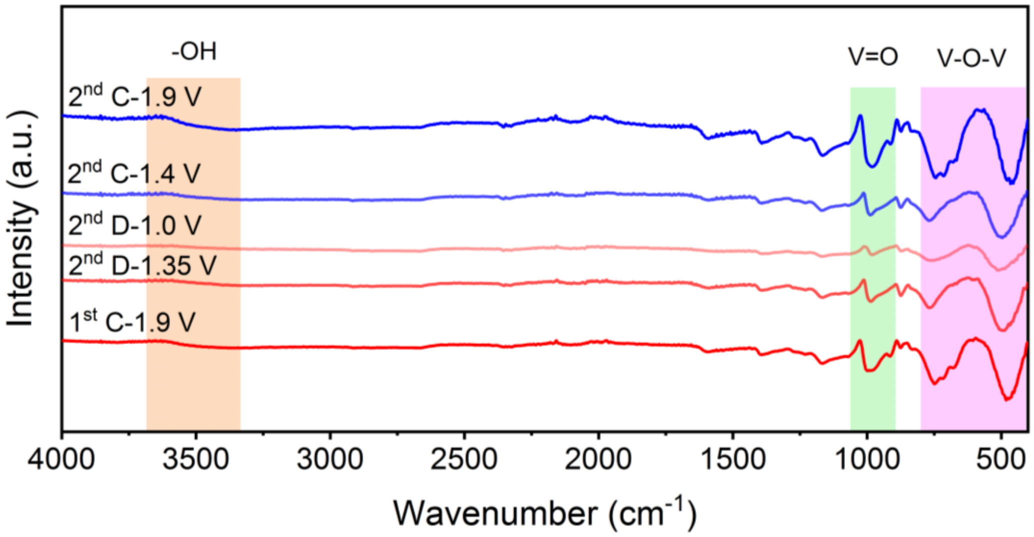

Supplementary Figure 19. FTIR spectra of MnVO cathodes at different charge/discharge states.

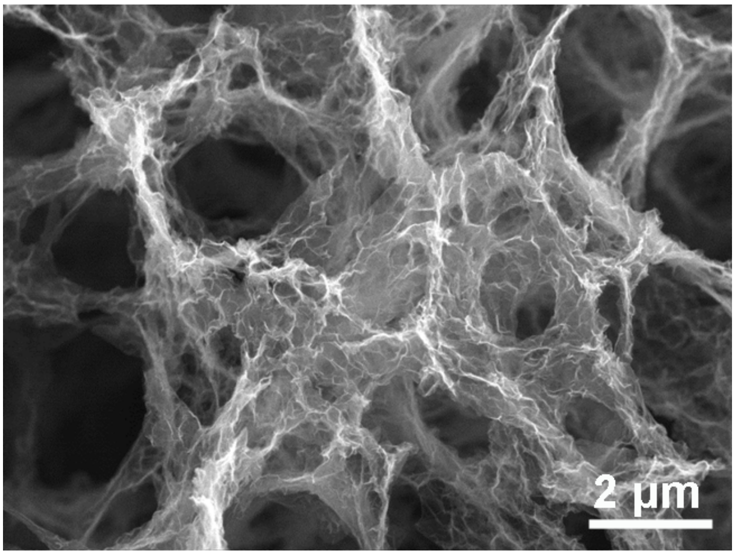

Supplementary Figure 20. SEM image of rGO foam.

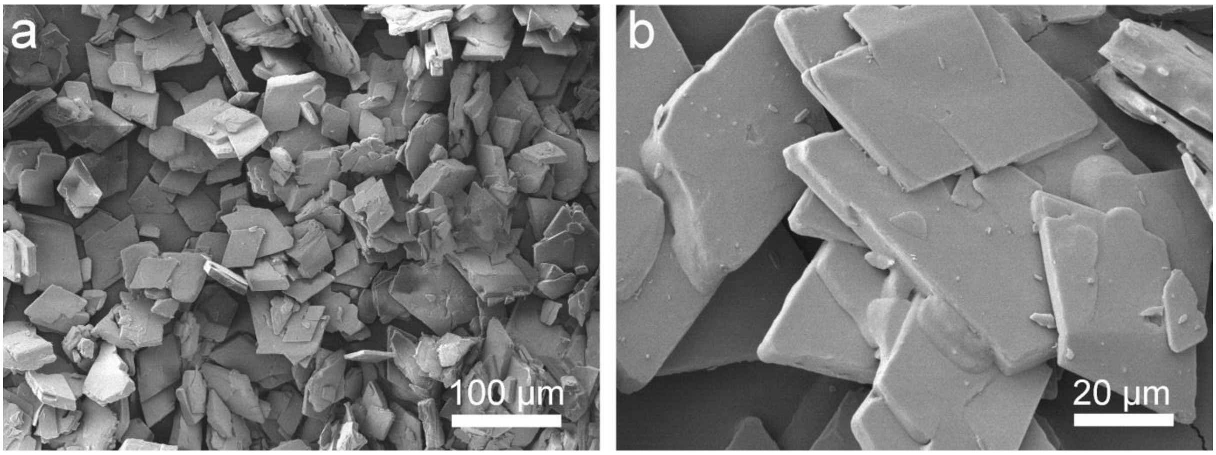

Supplementary Figure 21. a-b SEM images of commercial 4-Cl-BQ.

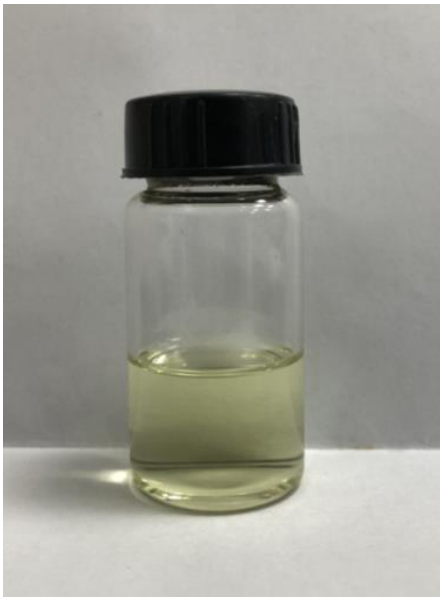

Supplementary Figure 22. Optical image of 4-Cl-BQ/$CHCl_3$ solution.

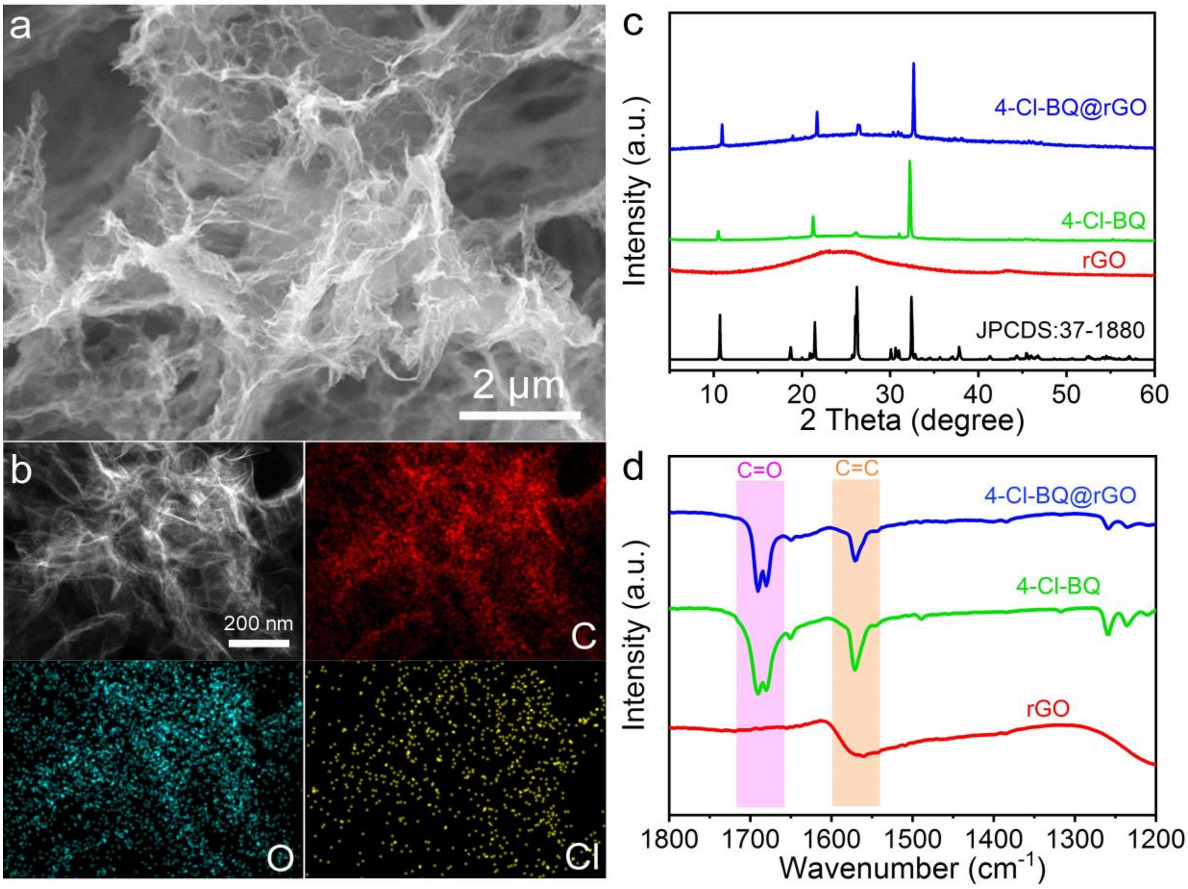

Supplementary Figure 23. a SEM image and **b** TEM elemental mapping of 4-Cl-BQ@rGO composites. **c** XRD patterns and **d** FTIR spectra of rGO, 4-Cl-BQ and 4-Cl-BQ@rGO composites.

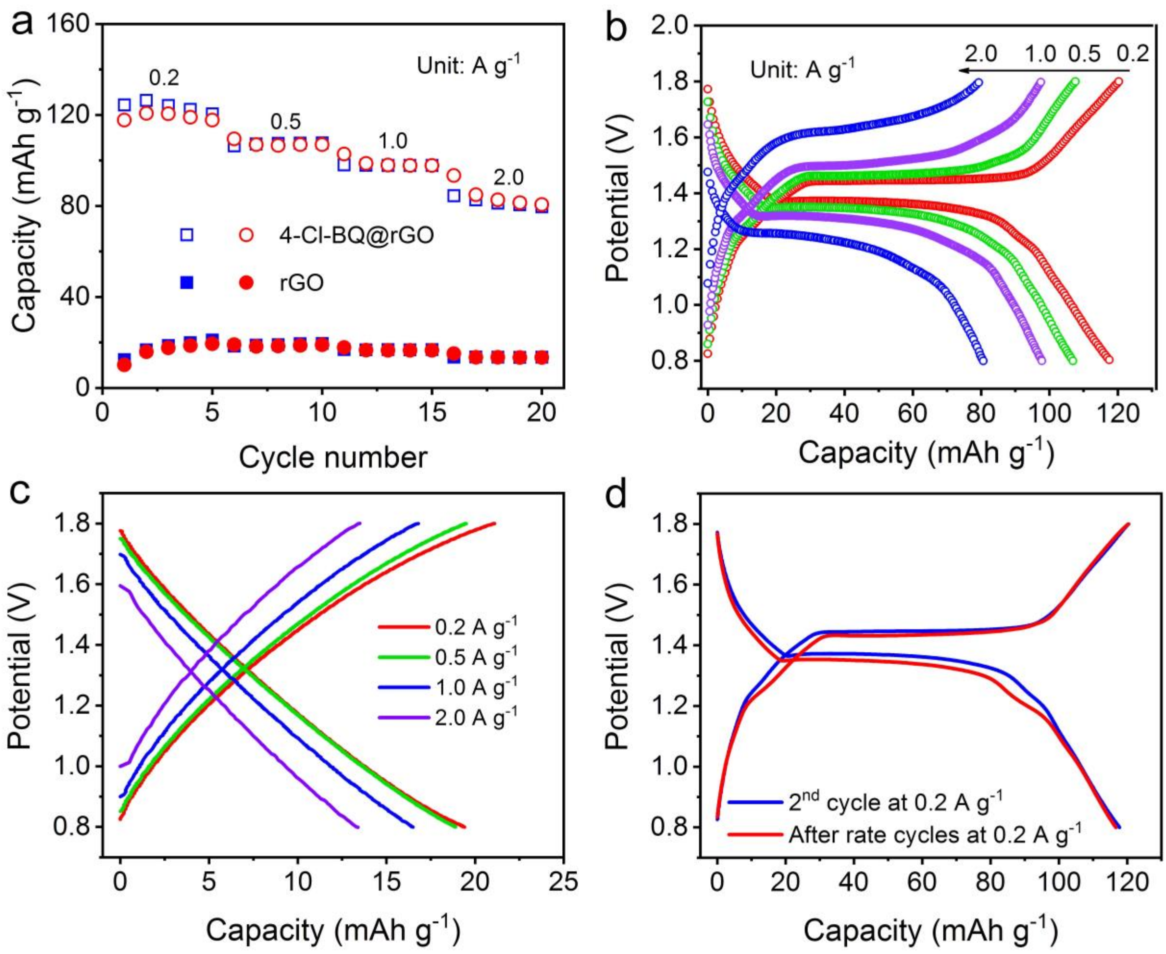

Supplementary Figure 24. a The rate performance and **b-c** corresponding GCD curves of Mn||4-Cl-BQ@rGO and Mn||rGO cells. **d** GCD curves of the Mn||4-Cl-BQ cell after rate cycles.

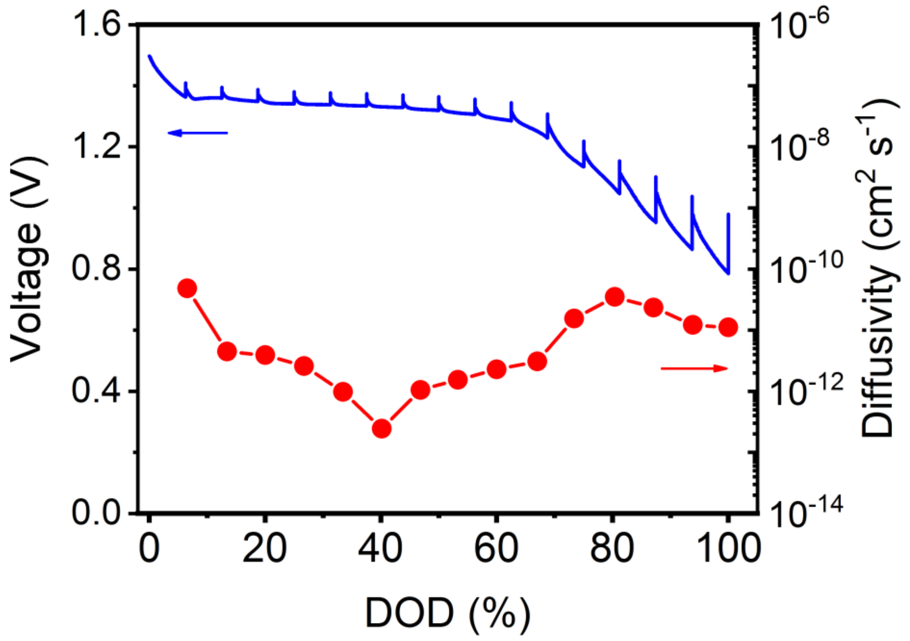

Supplementary Figure 25. GITT curve at 0.2 A g^{-1} and the D_{ion} of Mn||4-Cl-BQ cell.

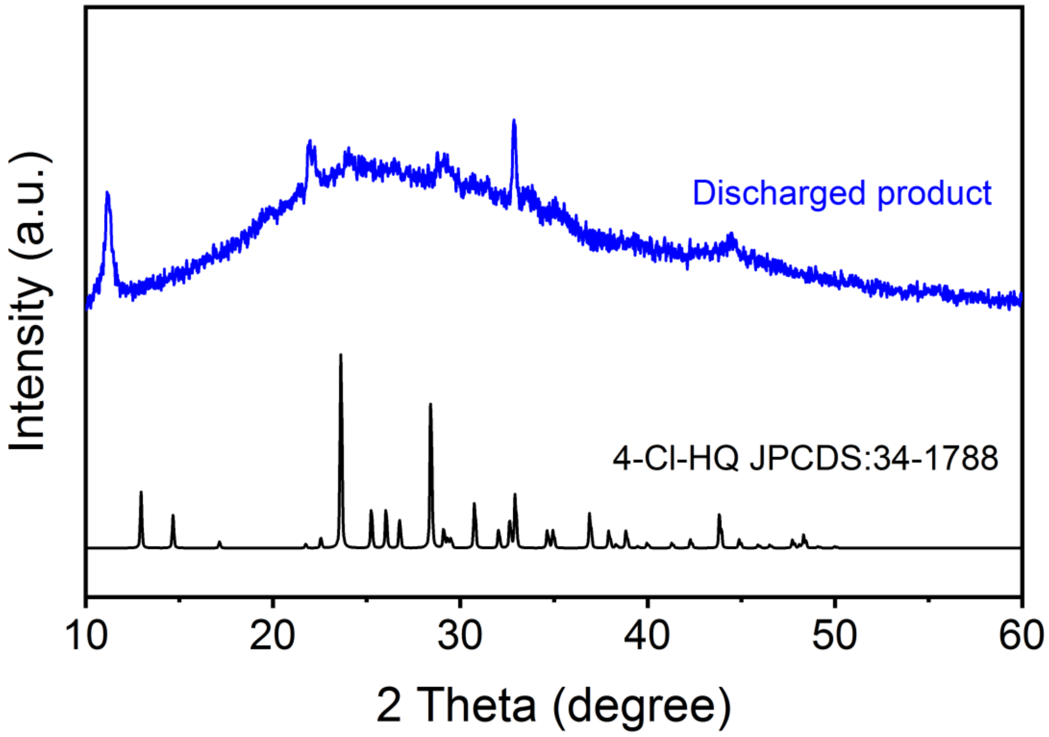

Supplementary Figure 26. XRD patterns of 4-Cl-HQ and the discharged product.

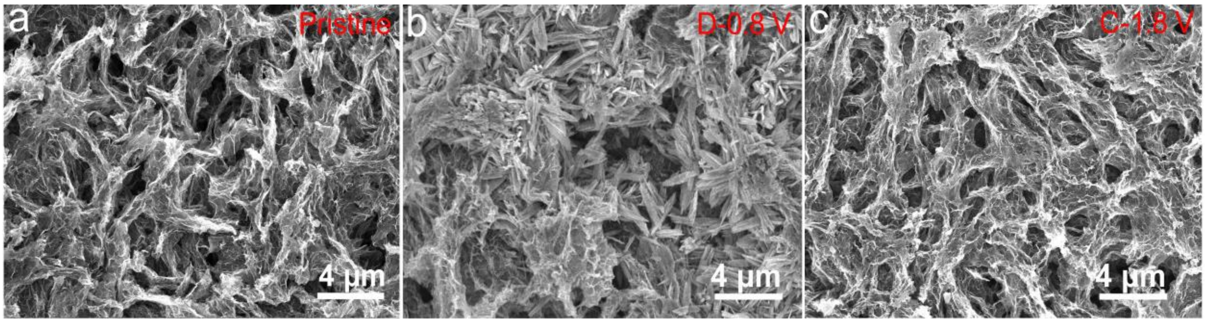

Supplementary Figure 27. SEM images of 4-Cl-BQ@rGO cathodes at **a** pristine, **b** discharged and **c** recharged states.

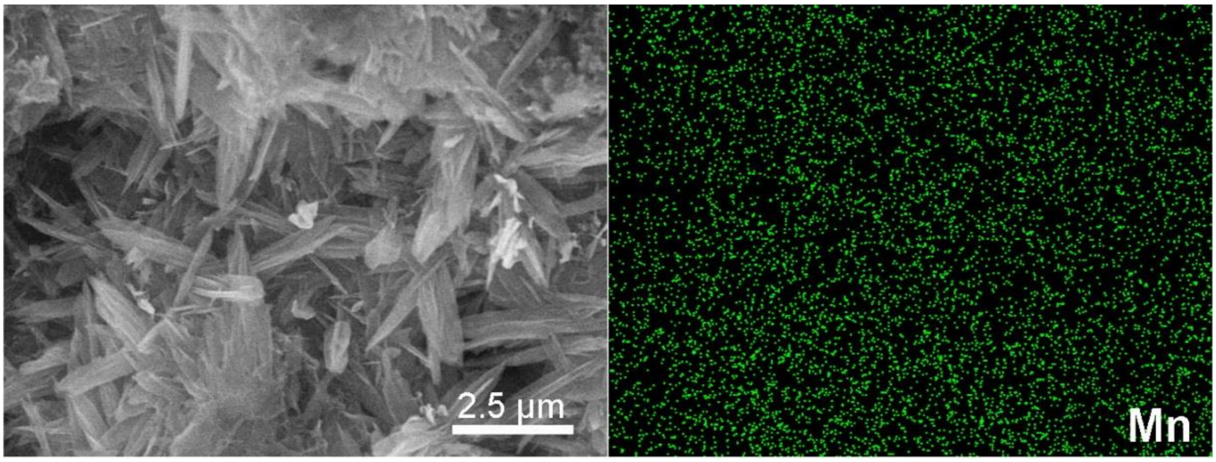

Supplementary Figure 28. Mn element mapping of the discharged products.

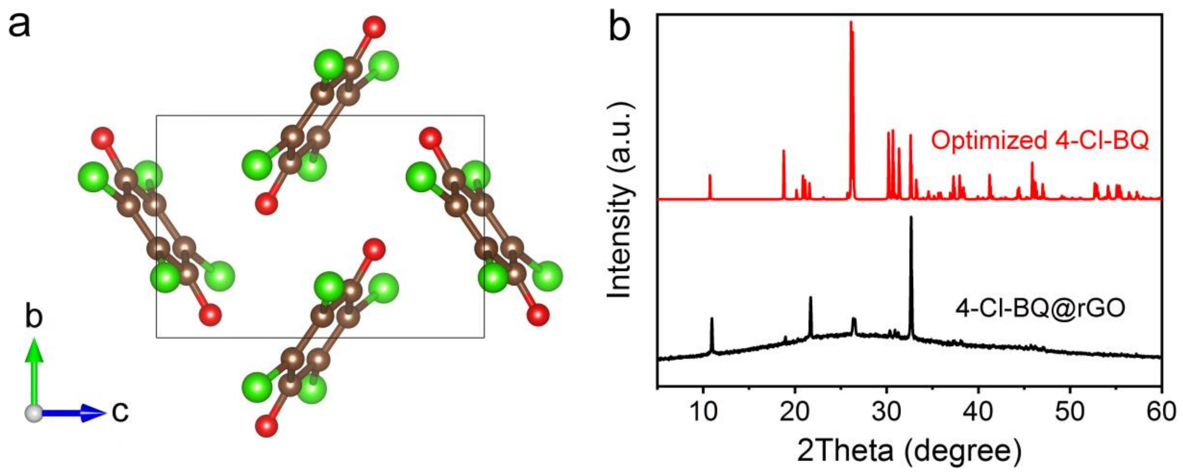

Supplementary Figure 29. a Models of simulated 4-Cl-BQ. **b** XRD patterns of optimized 4-Cl-BQ and 4-Cl-BQ@rGO electrodes.

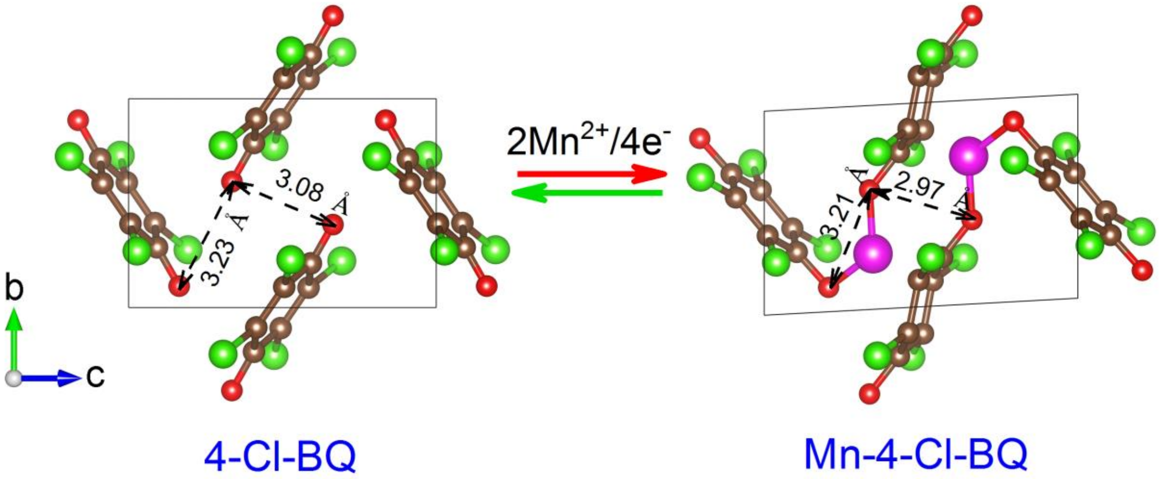

Supplementary Figure 30. Models of pristine 4-Cl-BQ and Mn^{2+} coordinated 4-Cl-BQ.

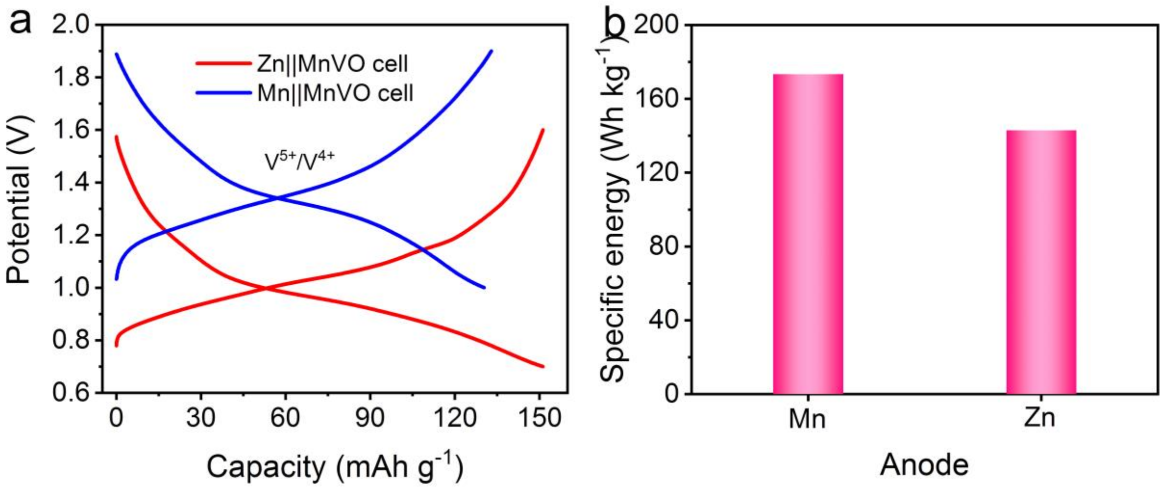

Supplementary Figure 31. a GCD curves of Mn||MnVO and Zn||MnVO cells based on V^{5+}/V^{4+} transition and **b** corresponding specific energy.

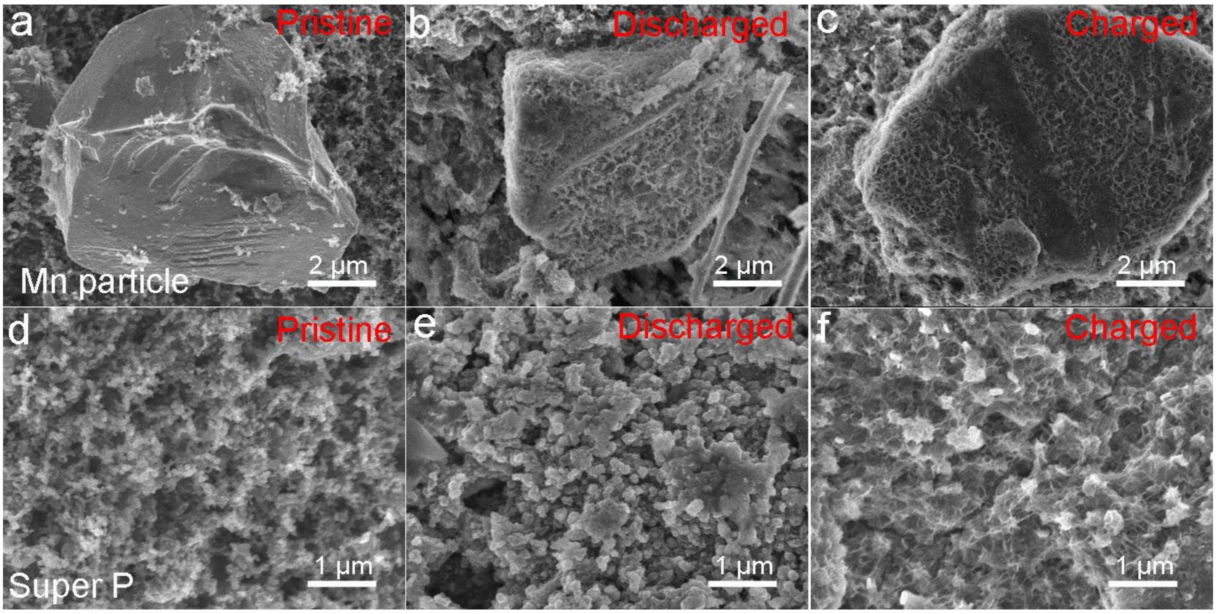

Supplementary Figure 32. a-f SEM images of Mn/C anode at different charge/discharge states.

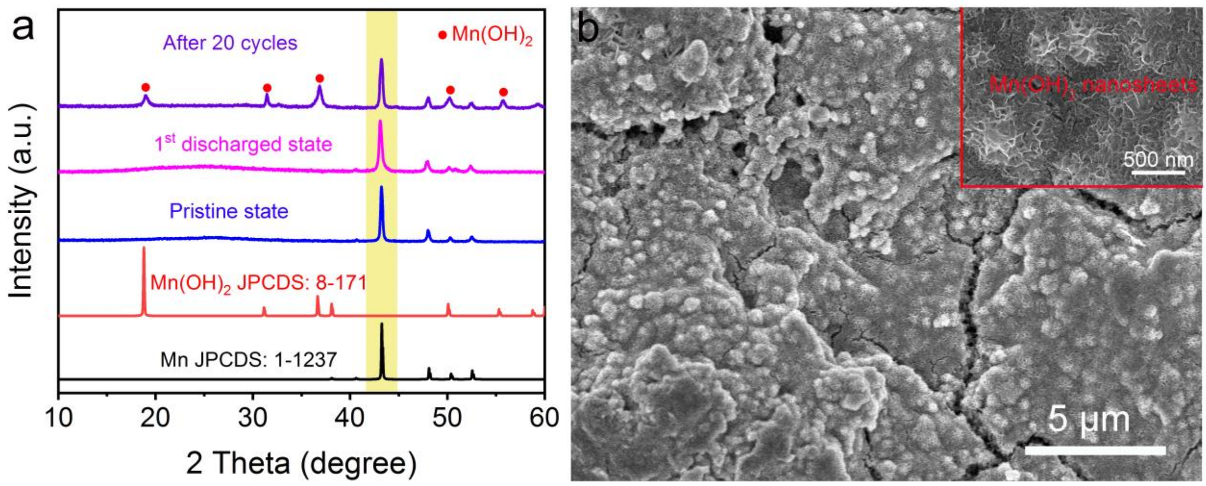

Supplementary Figure 33. a XRD patterns of Mn/C anodes at different charge/discharge states in Mn||MnVO cells. **b** SEM images of the Mn/C anode after 20 charge/discharge cycles.

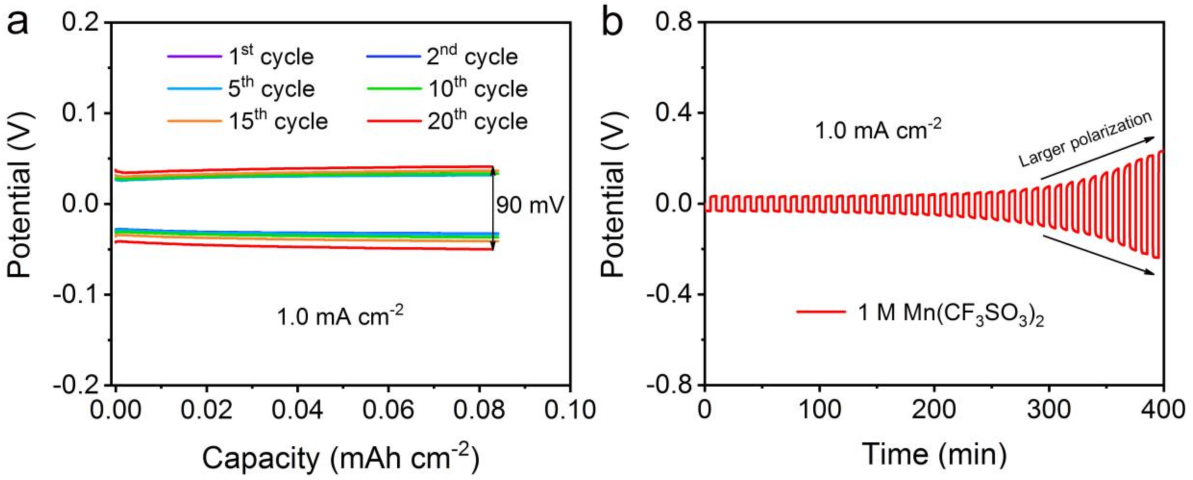

Supplementary Figure 34. a GCD curves and **b** cycling behavior of Mn/C||Mn/C symmetric cells at 1.0 mA cm^{-2}.

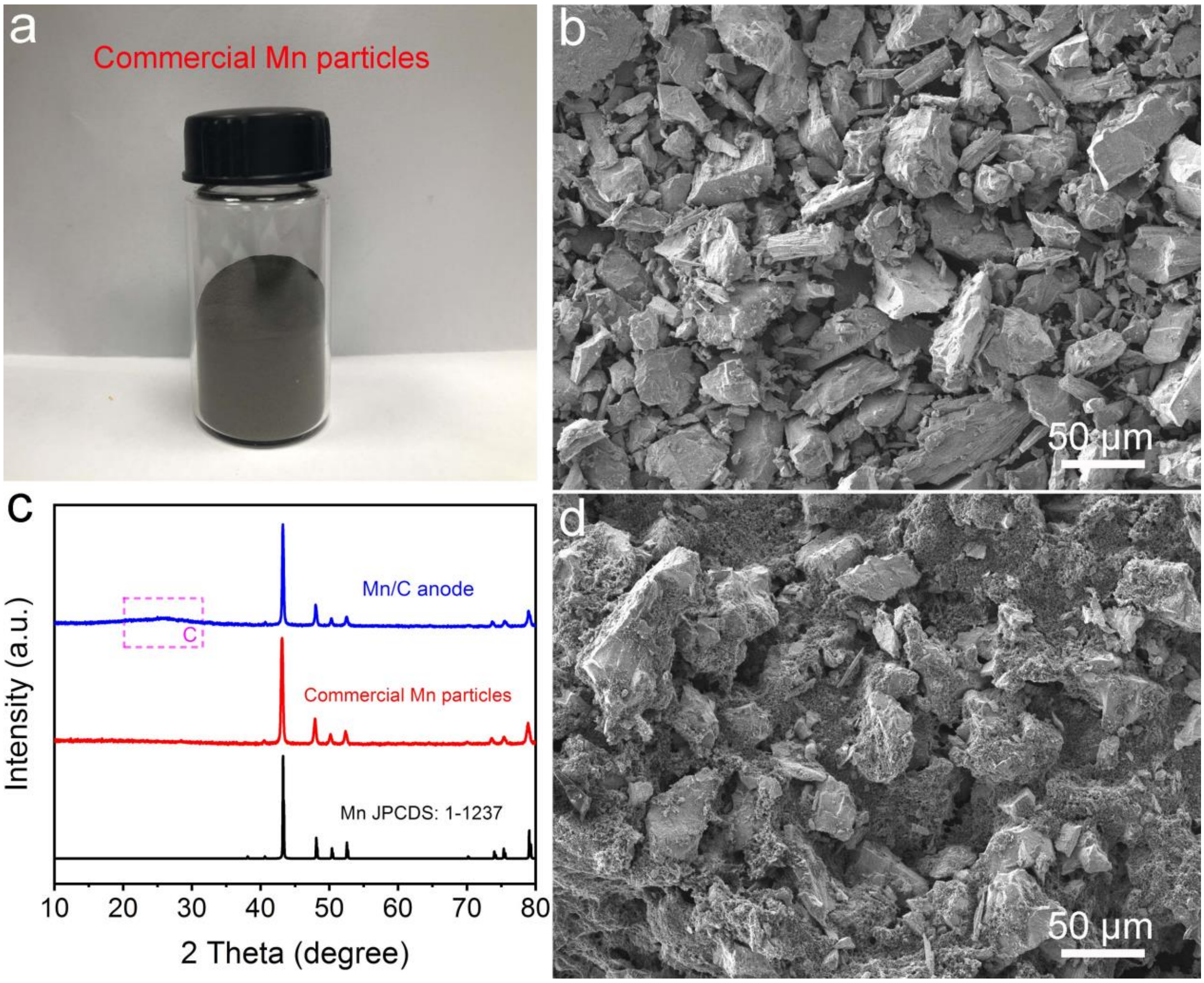

Supplementary Figure 35. a Optical image, **b** SEM image and **c** XRD patterns of commercial Mn metal and Mn/C composite anode. **d** SEM image of Mn/C composite anode.

Supplementary Tables

Supplementary Table 1. Fitted impendence parameters of Mn||MnVO cells.

Parameters (Ω)	$Mn(CF_3SO_3)_2$		$MnSO_4$	
	Value	Error (%)	Value	Error (%)
R_1	8.50	0.95	10.21	3.42
R_2	2.31	5.43	29.11	13.57
R_3	23.24	2.49	15.28	18.43
Z_w	1290	3.91	6640	6.24

Supplementary Table 2. ICP-AES analysis: Mn and V compositions in MnVO electrodes at different charge/discharge states (at 0.2 A g^{-1}).

Sample	Mn (μmol/L)	V (μmol/L)	Mn/V
Pristine	2.02	22.41	0.090
1st D-1.0 V	6.70	17.39	0.385
1st C-1.35 V	4.67	22.47	0.208
1st C-1.9 V	2.27	23.47	0.097
2nd D-1.4 V	4.57	19.92	0.230
2nd D-1.0 V	7.24	18.33	0.395

Supplementary Notes

Supplementary Note 1. Composition of MnVO nanosheets

ICP-AES study was employed to quantify the composition of MnVO, in which the molar ratio of Mn and V is 0.09 (Supplementary Table 2). Besides, the water content in MnVO is around 10% (Supplementary Figure 9), equivalent to ~1.18 water molecule in per formula unit. Therefore, the chemical composition of MnVO nanosheets is $Mn_{0.18}V_2O_5 \cdot 1.18H_2O$.

Supplementary Note 2. The transition of 4-Cl-BQ in dissolution/evaporation process

Commercial 4-Cl-BQ shows sheet morphology with tens of micrometers in size (Supplementary Figure 21). 4-Cl-BQ sheets were dissolved in $CHCl_3$ and then the resultant solution was dripped into rGO foams (Supplementary Figure 22). After drying, 4-Cl-BQ@rGO composites were obtained, which display similar porous structure with pristine rGO foams. 4-Cl-BQ with nanostructure is uniformly distributed on the surface of rGO sheets without observation of large particles, as suggested by SEM, TEM elemental mapping, FTIR and XRD results (Supplementary Figure 23).

Supplementary Note 3. Mn/C anode behaviors upon continuous cycling

To investigate the Mn/C anode behaviors upon continuous cycling, Mn/C||Mn/C symmetric cells were assembled and tested. The Mn/C anodes exhibit stable voltage profiles at initial 20 charge/discharge cycles (1.0 mA cm^{-2}) (Supplementary Figure 34a). However, after 20 cycles, the voltage hysteresis is fast increased (Supplementary Figure 34b), indicating the degradation of cycling performance.

Supplementary References

1 Liu, C. *et al.* Expanded hydrated vanadate for high-performance aqueous zinc-ion batteries. *Energy Environ. Sci.* **12**, 2273-2285 (2019).
